# Potential effects of β-hydroxy-β-methylbutyrate and exercise on brain aging and age-related disorders: implications for immunometabolic regulation and muscle–brain–immune crosstalk

**DOI:** 10.3389/fnagi.2026.1878831

**Published:** 2026-08-13

**Authors:** Boyuan Shi, Zheng Han

**Affiliations:** School of Physical Education, Soochow University, Suzhou, Jiangsu, China

**Keywords:** aging, brain–muscle–immune axis, endurance performance, molecular neurobiology, neuropsychiatric disorders, β-hydroxy-β-methylbutyrate

## Abstract

β-Hydroxy-β-methylbutyrate (HMB), a bioactive leucine metabolite, has emerged as a potentially valuable nutritional compound with effects extending beyond skeletal muscle metabolism toward systemic immunometabolic regulation. This review summarizes evidence from 24 experimental and clinical studies regarding the potential relevance of HMB to brain aging and age-related disorders, with emphasis on mitochondrial regulation, inflammatory signaling, redox homeostasis, and exercise-associated adaptations. Most studies report improvements in muscle strength, physical performance, and functional capacity, frequently without substantial increases in muscle mass. Proposed mechanisms include modulation of protein turnover, attenuation of apoptosis, regulation of autophagy, and preservation of mitochondrial dynamics. Emerging evidence also suggests that HMB may influence inflammatory gene expression, oxidative stress pathways, and immune-related signaling. However, direct central nervous system (CNS)-specific evidence remains limited, as most available findings derive from peripheral, preclinical, or exercise-associated models. Importantly, many included studies employed multicomponent interventions involving resistance training, creatine, vitamin D, or combined nutritional supplementation, making it difficult to isolate HMB-specific effects. Exercise itself appears to remain the principal driver of mitochondrial adaptation, inflammatory modulation, and functional improvement, whereas HMB demonstrates primarily adjunctive or context-dependent effects. Consequently, HMB should presently be regarded as a potential indirect modulator of muscle–brain–immune interactions during aging rather than a definitively established neuroactive compound. Further mechanistically rigorous and CNS-focused studies are required.

## 1 Introduction

β-Hydroxy-β-methylbutyrate (HMB) represents a bioactive metabolite that is synthesized from the essential branched-chain amino acid leucine (He et al., 2016). Approximately 5–10% of dietary leucine undergoes conversion into HMB through the intermediary *α*-ketoisocaproate (KIC), predominantly facilitated by the enzymatic action of KIC dioxygenase within the liver and various other tissues (Van Koevering and Nissen, 1992). While leucine is extensively acknowledged for its pivotal role in the activation of the mTOR signaling cascade and the facilitation of protein synthesis, HMB exhibits additional pleiotropic effects that surpass the traditional anabolic functions associated with its precursor (Wilkinson et al., 2013; Brioche et al., 2016). HMB has been demonstrated to modulate protein turnover through the stimulation of protein synthesis and the attenuation of proteolytic processes, partly through the modulation of the ubiquitin–proteasome pathway alongside autophagy-related signaling mechanisms (Girón et al., 2015). Furthermore, emerging evidence indicates that HMB exerts influences on mitochondrial biogenesis, cellular energy metabolism, and the responses to oxidative stress, suggesting a more comprehensive metabolic role that may not be entirely elucidated by leucine signaling alone (Wilkinson et al., 2013; Stancliffe, 2012). These attributes have established HMB as a viable nutritional intervention for conditions such as sarcopenia, cachexia, and the enhancement of exercise performance (Prado et al., 2022; Cruz-Jentoft, 2018). Despite comprehensive investigations, the predominant body of research concerning HMB has predominantly concentrated on skeletal muscle physiology, with an emphasis on hypertrophy, muscle preservation, and recuperation subsequent to exercise or disease-induced atrophy (Slater and Jenkins, 2000). This muscle-centric framework, while informative, embodies numerous constraints. Primarily, it neglects the systemic characteristics of metabolic regulation, wherein skeletal muscle engages in bidirectional communication with other organs, encompassing the brain and the immune system. Additionally, it does not consider the finding that HMB supplementation has the potential to enhance outcomes such as fatigue resistance, cognitive performance, and overall functional capacity (Eley et al., 2008; Townsend et al., 2015). Moreover, aging and neuropsychiatric disorders are typified by intricate, multisystem dysfunctions that involve mitochondrial dysfunction, persistent low-grade inflammation, and dysregulated neuroimmune communication (de Aguiar da Costa et al., 2026; Zhang et al., 2025). A solely muscle-centric paradigm is inadequate to elucidate how HMB may confer beneficial effects within these particular contexts. Consequently, there exists a pressing necessity to transcend reductionist frameworks and evaluate HMB through an integrative, systems biology lens.

Recent developments in the fields of molecular neurobiology and exercise physiology have illuminated the presence of a dynamic brain–muscle–immune axis, wherein metabolites, cytokines, and myokines facilitate intercommunication between peripheral tissues and the central nervous system (CNS) (Kong et al., 2025; Severinsen and Pedersen, 2020). Within this conceptual framework, HMB is posited as a prospective signaling molecule that may modulate not only muscle metabolism but also neuronal functionality, neuroinflammatory responses, and mitochondrial dynamics (Wilkinson et al., 2026; Belagodu et al., 2021). Empirical evidence indicates that HMB may exert influence over critical neurobiological processes, such as synaptic plasticity, microglial activation, and the regulation of oxidative stress, potentially via pathways that involve AMPK, mTOR, and NF-κB signaling cascades (Wilkinson et al., 2026; Liao, 2012; Liu et al., 2025). Moreover, mitochondrial dynamics which include processes of fission, fusion, and mitophagy, are increasingly acknowledged as pivotal to both muscular endurance and cerebral health. The dysregulation of these processes is a defining characteristic of aging as well as various neuropsychiatric disorders, which encompass depression, Alzheimer’s disease (AD), and Parkinson’s disease (PD).

By synthesizing these insights, a neurobiological paradigm for HMB offers a more holistic comprehension of its systemic ramifications, establishing connections among cellular energetics, inflammatory mechanisms, and inter-organ interactions. This perspective is especially pertinent for geriatric populations, wherein reductions in physical performance are closely associated with cognitive decline and immune system dysregulation. In this regard, the current review advocates for a conceptual transformation from a muscle-centric perspective of HMB towards a multidimensional framework that incorporates mitochondrial functionality, neuroinflammatory processes, and the brain–muscle–immune nexus. This integrative framework may elucidate innovative therapeutic avenues for augmenting endurance capabilities and alleviating neurodegenerative as well as neuropsychiatric conditions.

## 2 Literature search strategy

This narrative review was conducted through a comprehensive literature search in PubMed, Scopus, Web of Science, and Google Scholar databases for studies published up to January 2026. The search strategy included combinations of the following keywords: “β-hydroxy-β-methylbutyrate” OR “HMB” AND “exercise” OR “physical activity” AND “brain aging,” “neurodegeneration,” “sarcopenia,” “muscle–brain crosstalk,” “immunometabolism,” and “age-related diseases.”

Experimental, preclinical, and clinical studies investigating the effects of HMB and/or exercise on aging-related physiological and neurological outcomes were considered eligible. Articles not published in English, conference abstracts, duplicate publications, and studies not directly relevant to the scope of this review were excluded. The included studies were narratively synthesized to summarize current evidence regarding the potential immunometabolic and muscle–brain–immune regulatory effects of HMB and exercise. As this article is a narrative review, a formal systematic quality assessment or risk-of-bias analysis was not performed.

## 3 Molecular biochemistry and pharmacokinetics of HMB

### 3.1 Endogenous production and metabolism

HMB is synthesized endogenously from the essential branched-chain amino acid leucine through a highly regulated metabolic pathway. Initially, leucine undergoes reversible transamination mediated by branched-chain aminotransferase, resulting in the formation of KIC. Subsequently, a relatively minor proportion of KIC is converted into HMB through the enzymatic action of KIC dioxygenase, predominantly within the liver; however, the kidneys and skeletal muscle may also play a role in this conversion (Holeček, 2017). The majority of KIC, conversely, is directed towards mitochondrial oxidative decarboxylation via the branched-chain *α*-keto acid dehydrogenase complex, highlighting that while HMB production is quantitatively constrained, it remains biologically significant (Neinast et al., 2019; Shimomura et al., 2004). Following its synthesis, HMB can be metabolized through two predominant pathways. One pathway encompasses its transformation into β-hydroxy-β-methylglutaryl-CoA (HMG-CoA), a crucial intermediate in the biosynthesis of cholesterol, which is vital for the preservation of membrane integrity and repair within skeletal muscle and potentially in neuronal cells (Kandarian and Jackman, 2006). Alternatively, HMB may undergo oxidative processes to produce acetyl-CoA derivatives, thereby establishing its connection to mitochondrial energy production and cellular bioenergetics (Brioche et al., 2016). Significantly, the endogenous production of HMB appears to diminish with advancing age and under catabolic conditions such as sarcopenia, cancer cachexia, and chronic inflammation, which are characterized by dysfunctional leucine metabolism and mitochondrial impairment (Brioche et al., 2016; Cruz-Jentoft et al., 2019). This decline offers a biochemical basis for the administration of exogenous HMB supplementation as an intervention aimed at reestablishing metabolic homeostasis and protein equilibrium.

### 3.2 Cellular uptake and bioavailability

HMB is a diminutive, hydrophilic compound that is proficiently assimilated within the gastrointestinal tract subsequent to oral consumption. Two predominant supplemental variants have been extensively investigated: calcium HMB (Ca-HMB) and the free acid variant (HMB-FA), which exhibit notable distinctions in their pharmacokinetic characteristics. HMB-FA demonstrates accelerated absorption kinetics, elevated peak plasma concentrations (C_max), and enhanced tissue bioavailability relative to Ca-HMB, presumably attributable to its superior solubility and intestinal permeability (Fuller et al., 2011; Churchward-Venne et al., 2014). Peak plasma concentrations of HMB are generally attained within a time frame of 30–60 min for HMB-FA and 1–2 h for Ca-HMB, with a plasma half-life estimated to be approximately 2–3 h (Fuller et al., 2011; Wilson et al., 2013). HMB circulates predominantly in its unbound form and is internalized by tissues through transport mechanisms that remain inadequately defined but are postulated to involve monocarboxylate transporters (Juel, 1997). At the cellular level, HMB modulates multiple anabolic and catabolic signaling pathways. The activation of mTORC1 signaling facilitates the enhancement of protein synthesis while concurrently inhibiting proteolysis through the downregulation of the ubiquitin–proteasome system, which encompasses the suppression of muscle-specific E3 ligases such as MuRF1 and atrogin-1 (Eley et al., 2008; Bodine and Baehr, 2014). Furthermore, HMB has been demonstrated to modulate AMPK signaling and to promote mitochondrial biogenesis through the upregulation of PGC-1α, thereby establishing a connection to enhanced oxidative metabolism and improved endurance capacity (He et al., 2016). Moreover, HMB possesses notable antioxidant and anti-inflammatory characteristics, which include the reduction of ROS production and the inhibition of NF-κB signaling, thereby implying a significant role in cellular stress resistance that extends beyond mere muscle anabolism (Shakibaee et al., 2023; Smith et al., 2005).

### 3.3 Tissue-specific distribution (muscle vs. brain hypothesis)

**Figure 1:**
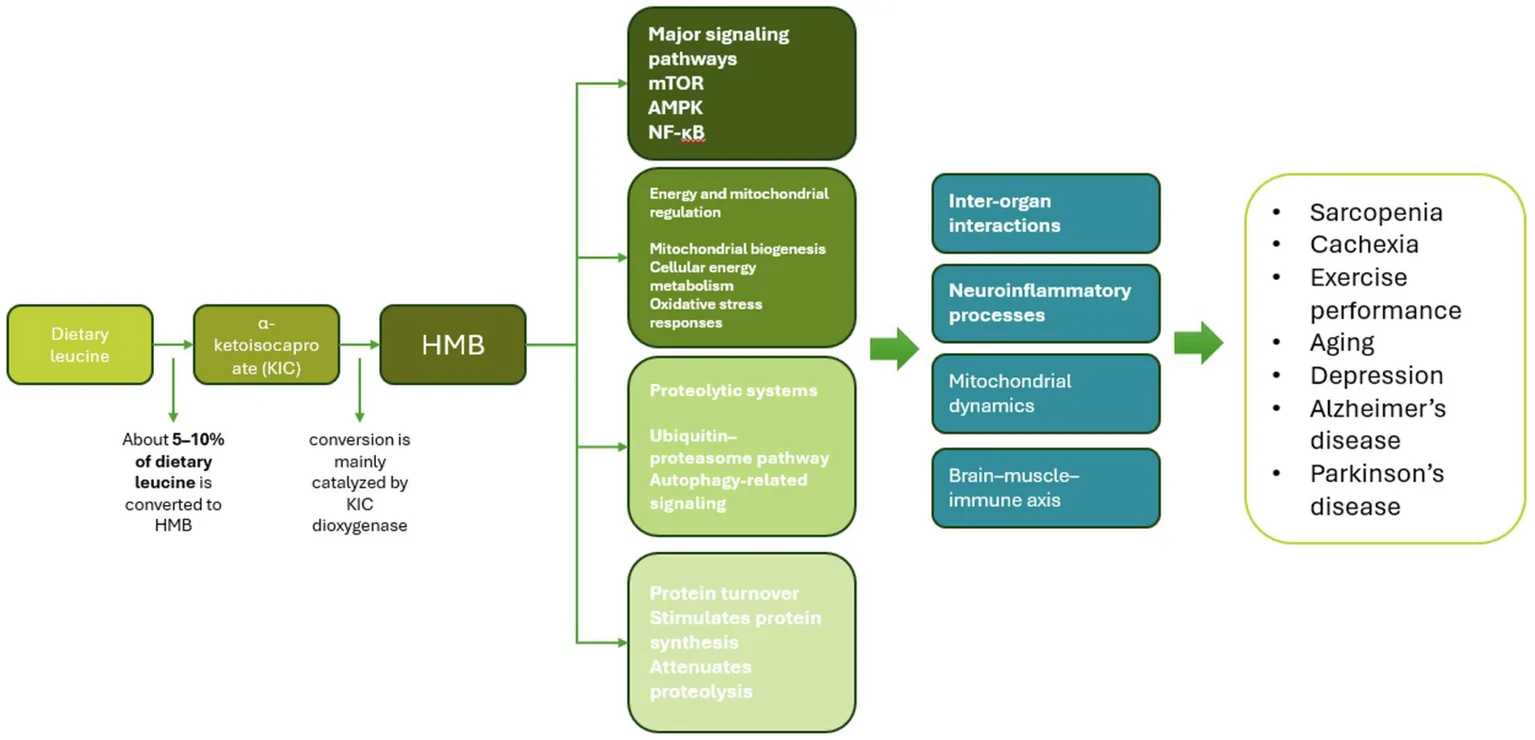
Molecular biochemistry and pharmacokinetics of HMB.

The distribution of HMB within various tissues has been most thoroughly elucidated in skeletal muscle, where it accrues to manifest anabolic, anti-catabolic, and cytoprotective properties. Within muscular tissue, HMB plays a pivotal role in maintaining sarcolemmal integrity, augments mitochondrial oxidative capacities, and facilitates recovery subsequent to exercise-induced muscular damage (Wilson et al., 2013). Its metabolic conversion to HMG-CoA may promote local cholesterol biosynthesis, which is essential for the repair of membranes and the maintenance of structural integrity. Nonetheless, an increasing scholarly focus has been allocated to the prospective distribution and functional roles of HMB within the CNS. Although empirical quantitative data regarding HMB concentrations in cerebral tissue remain sparse, its physicochemical characteristics and structural resemblance to short-chain fatty acids and derivatives of ketone bodies imply that it may traverse the blood–brain barrier (BBB) through monocarboxylate transporters, particularly MCT1, which are expressed in endothelial cells and astrocytes (Pierre and Pellerin, 2005). This has precipitated the development of the “muscle versus brain hypothesis,” which asserts that the systemic effects of HMB are facilitated through both peripheral (muscle) and central (brain) pathways. Within the CNS, HMB may modulate neuronal energy metabolism, mitochondrial dynamics, and neuroinflammatory processes. Considering that mitochondrial dysfunction and chronic neuroinflammation are characteristic features of aging and various neuropsychiatric disorders (Aunan et al., 2016), such as AD, PD, and major depressive disorder, HMB may confer neuroprotective effects either directly or indirectly. Preclinical investigations further corroborate this integrative framework, indicating that HMB supplementation may mitigate neuroinflammation, diminish oxidative stress, and enhance cognitive performance under pathological circumstances (Belagodu et al., 2021). Although empirical evidence from human studies is still in its nascent stages, these observations provide a compelling justification for the consideration of HMB as a multi-organ metabolic regulator with significant implications for neurobiology (Figure 1).

Dietary leucine is converted to HMB via *α*-ketoisocaproate (KIC), with approximately 5–10% of leucine undergoing this conversion, primarily through KIC dioxygenase activity in the liver and other tissues. HMB modulates protein turnover by stimulating protein synthesis and suppressing proteolysis, in part through effects on the ubiquitin–proteasome and autophagy-related pathways. It also influences mitochondrial biogenesis, cellular energy metabolism, oxidative stress responses, and signaling pathways including mTOR, AMPK, and NF-κB. These molecular actions contribute to broader effects across the brain–muscle–immune axis, mitochondrial dynamics, and inter-organ communication, with relevance to sarcopenia, cachexia, exercise performance, aging, depression, Alzheimer’s disease, and Parkinson’s disease.

## 4 HMB and skeletal muscle signaling: beyond hypertrophy

### 4.1 Protein synthesis vs. proteolysis (mTOR, ubiquitin–proteasome)

HMB exerts a modulatory influence on skeletal muscle homeostasis through a bifunctional regulatory mechanism that encompasses both protein synthesis and degradation processes. HMB engages the mTORC1 signaling cascade, which facilitates the enhancement of translation initiation and ribosomal biogenesis, thus fostering the synthesis of muscle proteins. Simultaneously, it attenuates proteolytic activity by obstructing critical elements of the ubiquitin–proteasome pathway, specifically the E3 ubiquitin ligases MuRF1 and atrogin-1, which are known to be elevated during catabolic conditions. This anti-catabolic effect is partially mediated through the suppression of FOXO transcription factors and the NF-κB signaling pathway. Notably, the ramifications of HMB extend beyond mere anabolic stimulation, as it fortifies muscle cell integrity and mitigates inflammation-induced proteolytic signaling. This integrated regulation of both synthesis and degradation is particularly pertinent in the contexts of aging and disease, wherein anabolic resistance and heightened proteolysis coexist, thereby implying that HMB serves to restore the equilibrium of protein turnover rather than solely augmenting muscle mass (Wilkinson et al., 2013; Eley et al., 2008; Bodine and Baehr, 2014).

### 4.2 Functional performance vs. muscle mass paradox

Although HMB is extensively linked with muscle hypertrophy, a growing body of evidence elucidates a “functional performance versus muscle mass paradox,” wherein enhancements in strength, endurance, and recovery manifest independently of substantial increases in muscle mass. This indicates that HMB augments muscle quality (MQ) rather than muscle quantity. Mechanistically, HMB enhances mitochondrial efficiency, mitigates oxidative stress, and amplifies energy production, resulting in improved fatigue resistance and contractile performance. Furthermore, HMB may modulate excitation–contraction coupling and calcium handling, which are essential for effective force generation. These adaptations hold particular significance in aging populations, where gains in muscle mass are frequently constrained, yet functional enhancements are of clinical relevance. Consequently, the ergogenic effects of HMB are likely derived from integrated metabolic and cellular adaptations rather than hypertrophy in isolation, thus advocating for a paradigm shift towards the assessment of muscle function as a primary outcome (Brioche et al., 2016; Holeček, 2017; Wilson et al., 2013).

### 4.3 Neuromuscular adaptations

**Figure 2:**
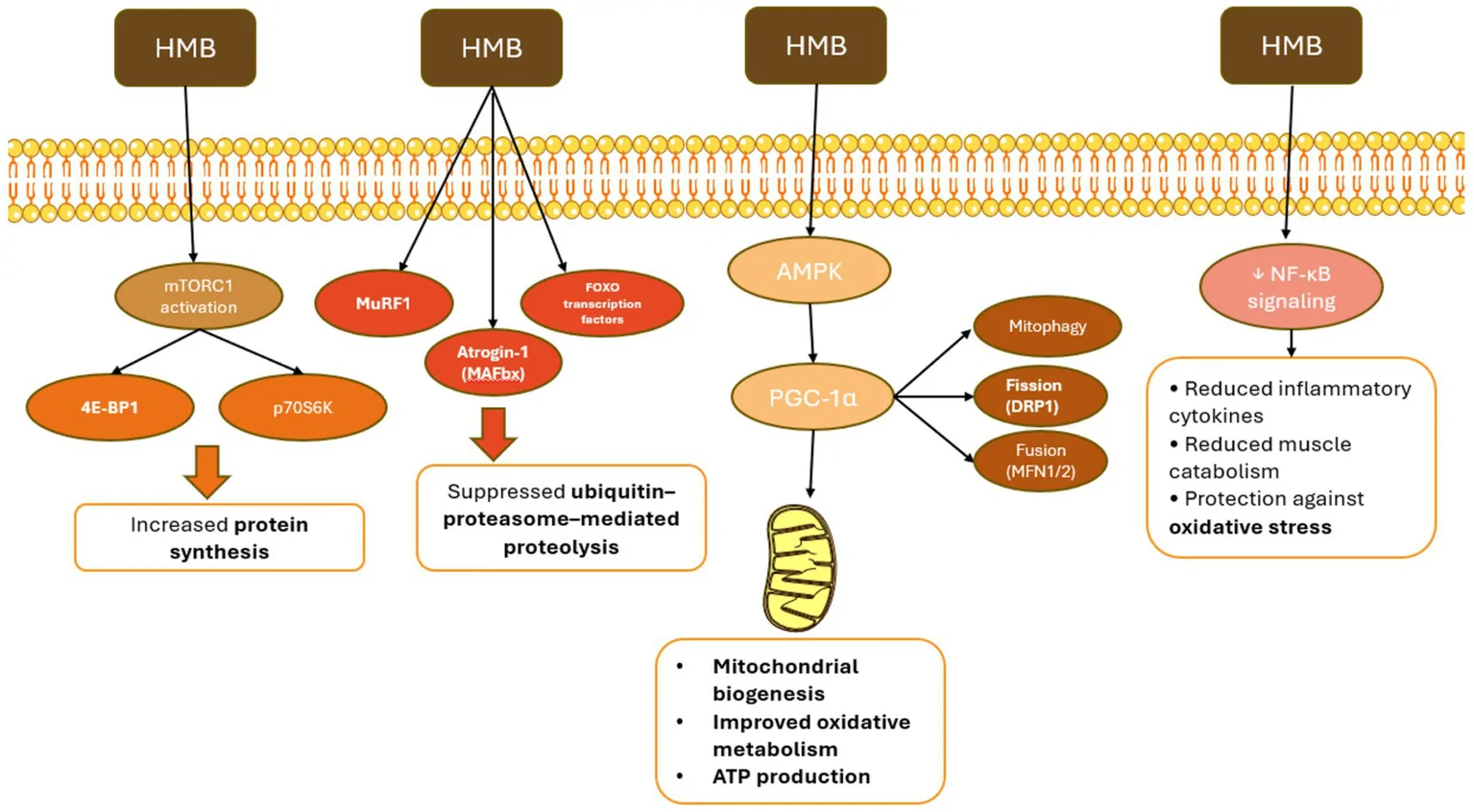
HMB-mediated skeletal muscle signaling beyond hypertrophy.

Beyond its metabolic implications, HMB may play a significant role in facilitating neuromuscular adaptations that improve motor function and coordination. Emerging research indicates that HMB may affect the integrity and functionality of the neuromuscular junction (NMJ), potentially through the stabilization of membrane structures and the enhancement of synaptic signaling. Additionally, HMB may indirectly augment motor unit recruitment and firing efficiency by enhancing muscle energetics and alleviating fatigue. Moreover, its anti-inflammatory and antioxidant characteristics could serve to protect peripheral nerves and NMJ components from degeneration, an aspect that holds particular significance in the context of aging and neuromuscular disorders. There is an increasing interest in the role of HMB in the modulation of myokine release, which may impact neural plasticity and motor control via the muscle–brain axis. Collectively, these findings imply that HMB supports neuromuscular performance through a synergistic influence on muscle, nerve, and synaptic function, rather than through isolated muscular adaptations (Eley et al., 2008; Courel-Ibáñez et al., 2019; Fernández-Landa et al., 2024) (Figure 2).

HMB modulates intracellular signaling networks within skeletal muscle fibers. Activation of mTORC1 enhances protein synthesis, while suppression of FOXO-regulated ubiquitin ligases such as MuRF1 and atrogin-1 reduces proteolysis. HMB also influences mitochondrial homeostasis through AMPK-PGC-1α signaling, promoting mitochondrial biogenesis, oxidative metabolism, and mitochondrial quality control mechanisms including fusion, fission, and mitophagy. Additionally, HMB attenuates NF-κB–mediated inflammatory signaling and oxidative stress pathways. These combined effects contribute to functional adaptations including improved muscle metabolism, enhanced endurance capacity, and protection against muscle wasting in conditions such as aging, sarcopenia, and cachexia.

## 5 Mitochondrial dynamics and bioenergetic remodeling

### 5.1 Evidence from preclinical and clinical studies

An investigation evaluated whether HMB supplementation enhances the effects of resistance training on MQ in older women at risk of sarcopenia. In this randomized, double-blind, placebo-controlled trial, women aged 65–79 years with low muscle mass received resistance training or educational intervention combined with either HMB or placebo for 12 weeks. Functional MQ (knee strength relative to muscle thickness) and compositional MQ (muscle echo intensity) were assessed. The results demonstrated no significant interaction between HMB and resistance training for either functional or compositional MQ, whereas resistance training alone significantly attenuated declines in functional MQ compared with the educational intervention. Importantly, neither resistance training nor HMB significantly altered compositional MQ, indicating limited evidence for HMB-specific improvements in muscle architecture or mitochondrial-related MQ outcomes in this cohort (Osuka et al., 2022) (Table 1). Another study investigated the molecular effects of HMB during bed rest and subsequent resistance training in older adults. Nineteen participants aged 60–76 years underwent 10 days of bed rest followed by 8 weeks of resistance training while receiving either HMB (3 g/day) or placebo. Muscle biopsies were analyzed for mitochondrial markers, autophagy-related proteins, atrophy signaling, and lipid composition. Bed rest increased protein degradation markers and mitochondrial complex I content in both groups, whereas HMB supplementation was associated with greater intramuscular triglyceride accumulation during inactivity. During rehabilitation, resistance training increased mitochondrial complex II expression in both groups, while HMB supplementation was associated with preservation of mitochondrial dynamics-related proteins, including DRP1 and MFN2. However, most improvements in autophagy and atrophy markers occurred similarly in both groups following resistance training, suggesting that exercise rather than HMB represented the dominant driver of mitochondrial and metabolic recovery (Standley et al., 1985).

**Table 1:** Effects of HMB supplementation on muscle quality, regeneration, mitochondrial function, immunometabolism, and body composition in aging models.

Population/model	Intervention	HMB formulation	Duration	Key outcomes	Findings	Reference
Clinical studies						
Older women (65–79 yr) with low muscle mass (SMI < 5.7 kg/m^2^)	RT + HMB (1,500 mg/day), RT + placebo, Education + HMB, Education + placebo	NA	12 weeks	Functional MQ (strength/quadriceps thickness); Compositional MQ (echo intensity)	No interaction between HMB and RT for MQ. RT attenuated decline in functional MQ, but HMB provided no additional benefit. No effect on compositional MQ.	Osuka et al. (2022)
Older adults (60–76 yr)	HMB (3 g/day) vs. control during 10-day bed rest + 8-week RT rehabilitation	Ca-HMB	~10 weeks total	Mitochondrial content (OXPHOS), dynamics (MFN2, DRP1), autophagy (BNIP3, LC3B), lipids	HMB maintained higher mitochondrial content and dynamics during rehabilitation. Increased intramuscular lipid storage during bed rest. No major mitochondrial changes during bed rest alone.	Standley et al. (1985)
Older adults (≥60 yr) with sarcopenia	HMB + RT vs. placebo + RT	NA	12 weeks	Strength, gait speed, MQ, inflammation, body composition	HMB significantly improved strength, physical performance, MQ, and reduced inflammatory markers; no change in muscle mass.	Yang et al. (2023)
Older adults with sarcopenia	Exercise ± HMB/nutritional supplementation	NA	12 weeks	T-cell inflammatory gene expression, leg strength	Combined HMB + exercise altered T-cell gene expression (e.g., IL-7R, PRKCQ) and correlated with improved muscle strength.	Ma et al. (2021)
Elderly men (66–78 yr)	RT ± HMB vs. controls	NA	12 weeks	Abdominal fat mass (DXA)	RT + HMB significantly reduced abdominal fat vs. all other groups; no effect with HMB alone.	Stout et al. (2015)
Preclinical studies						
Aged male rats (20 months) with voluntary wheel running	HMB + β-alanine vs. control diet	NA	8 weeks	Muscle force, fatigue resistance, CSA, mitochondrial (PGC-1α), autophagy (LC3B)	No improvement in force or fatigue resistance. Trend toward increased CSA but reduced muscle quality (force/CSA). Decreased PGC-1α and autophagy markers. Minimal functional benefit.	Russ et al. (2017)
Aged female rats (19 months)	RT ± HMB (0.48 g/kg/day)	NA	10 weeks	Strength, CSA, satellite cells, myonuclei, myogenic markers	RT increased strength, CSA, and satellite cells; HMB did not further enhance hypertrophy or myogenic capacity despite ↑IGF-I in soleus.	Kim et al. (2012)
Aging SAMP8 mice	Exercise ± HMB, ± black ginger, or combination	NA	16 weeks	Muscle mass, strength, mitochondrial genes, autophagy	Only combination (HMB + BG + exercise) improved muscle mass/strength; ↑ mitochondrial biogenesis and autophagy markers.	Aoki et al. (2022)

HMB, β-hydroxy-β-methylbutyrate; RT, resistance training; MQ, muscle quality; CSA, cross-sectional area; OXPHOS, oxidative phosphorylation; PGC-1α, peroxisome proliferator-activated receptor gamma coactivator 1-alpha; DXA, dual-energy X-ray absorptiometry; BG, black ginger. NA: Not Available.

### 5.2 Critical synthesis and translational considerations

The extant body of evidence pertaining to HMB-mediated mitochondrial and bioenergetic modifications is characterized by its heterogeneity and dependence on contextual factors. Although certain investigations indicate that HMB may contribute to the preservation of mitochondrial-associated proteins and facilitate aspects of mitochondrial remodeling during the recovery phase following disuse, these influences are typically modest and do not consistently translate into quantifiable enhancements in MQ or functional outcomes. Notably, resistance training has consistently emerged as the principal catalyst for mitochondrial and functional adaptations, whereas the supplementary effects of HMB exhibit variability across diverse studies. A multitude of methodological considerations may partially elucidate these discrepancies, including small sample sizes, brief intervention durations, variations in participant health statuses, heterogeneity in exercise protocols, and a reliance on indirect molecular biomarkers instead of standardized functional mitochondrial evaluations. Furthermore, certain observed molecular modifications, such as alterations in mitochondrial protein expression or lipid accumulation, may not necessarily correlate with clinically significant physiological advancements. These empirical observations collectively indicate that HMB may exert beneficial or modulatory influences on mitochondrial dynamics and bioenergetic remodeling under specific physiological conditions, particularly during the recovery phase from inactivity or catabolic stress. Nevertheless, the existing body of evidence does not consistently substantiate significant or independent mitochondrial advantages beyond those elicited by exercise alone. Moreover, any translational interpretations should be approached with caution, as the majority of the currently accessible evidence is derived from investigations focused on peripheral skeletal muscle rather than direct studies oriented towards CNS bioenergetics.

### 5.3 Molecular mechanisms underlying mitochondrial remodeling

Collectively, these studies indicate that the effects of HMB on mitochondrial remodeling are modest, heterogeneous, and highly context dependent. Although HMB may preserve selected mitochondrial dynamics-related proteins such as DRP1 and MFN2 and modestly influence OXPHOS-associated content during recovery from disuse, these effects are generally observed in combination with resistance training or rehabilitation protocols, making it difficult to distinguish HMB-specific actions from exercise-mediated adaptations. Some studies additionally reported increased intramuscular triglyceride accumulation during bed rest; however, the physiological significance of this finding remains unclear and may not necessarily reflect improved mitochondrial efficiency. Although HMB has been associated with increases in IGF-I expression in selected muscle groups, evidence supporting consistent enhancement of satellite cell activation, myonuclear accretion, or myofiber hypertrophy remains limited. Similarly, findings related to autophagy and mitochondrial biogenesis remain inconsistent across studies and experimental models. In some preclinical settings, HMB supplementation was even associated with reduced markers of mitochondrial biogenesis and autophagy, highlighting the complexity of its regulatory effects. Importantly, many reported mitochondrial and bioenergetic adaptations are also well-established consequences of RT itself, which remains the primary regulator of mitochondrial remodeling, neuromuscular adaptation, and metabolic recovery. Therefore, current evidence does not support strong independent mitochondrial effects of HMB beyond those induced by exercise interventions. In summary, available evidence suggests that HMB may exert limited adjunctive effects on mitochondrial dynamics, protein turnover, and metabolic regulation, particularly during recovery from disuse or catabolic stress. However, the magnitude and translational significance of these effects remain uncertain due to small sample sizes, heterogeneous study designs, and the frequent coexistence of exercise-based interventions.

## 6 Neuroimmune modulation, inflammatory signaling, autophagy and apoptosis

### 6.1 Clinical evidence

A randomized, double-blind, placebo-controlled study evaluated the effects of HMB supplementation combined with resistance training in older adults (≥60 years) with sarcopenia. Participants received either HMB or placebo twice daily for 12 weeks while participating in biweekly exercise sessions. The HMB group demonstrated greater improvements in handgrip strength, gait velocity, chair-stand performance, and MQ compared with placebo. In addition, circulating levels of tumor necrosis factor-like weak inducer of apoptosis, an inflammatory biomarker associated with muscle catabolism, were reduced in the HMB group. However, no significant differences were observed in muscle mass or overall body composition between groups, suggesting that the primary benefits were functional rather than hypertrophic. Importantly, because all participants underwent resistance training, it remains difficult to distinguish HMB-specific anti-inflammatory effects from exercise-mediated adaptations, which are themselves potent modulators of inflammatory signaling and neuromuscular function (Yang et al., 2023) (Table 1). Another study investigated the effects of exercise and nutritional supplementation on T-cell gene expression in older adults with sarcopenia. Participants were assigned to exercise alone, exercise plus supplementation, or control groups for 12 weeks. Both intervention groups improved lower-limb strength. The combined intervention additionally altered the expression of nine inflammation-related genes in T cells, while seven genes, including RASGRP1, LEF1, and IL-7R, correlated with increases in leg strength. These findings support the possibility that combined nutritional and exercise interventions may influence immune-related signaling pathways; however, the study design does not allow attribution of these molecular effects specifically to HMB because supplementation was administered concurrently with exercise and other nutritional components (Ma et al., 2021). Another investigation assessed the effects of 12 weeks of HMB supplementation combined with resistance training on abdominal adiposity in older men aged 66–78 years. Participants were assigned to placebo without training, HMB alone, resistance training with placebo, or resistance training combined with HMB. A significant reduction in abdominal fat mass was observed only in the resistance training + HMB group, whereas neither HMB nor resistance training alone produced significant changes relative to controls. These findings suggest a possible additive interaction between HMB and exercise; however, the absence of independent effects in the HMB-only group indicates that resistance training likely represented the principal driver of body composition changes. Therefore, the available evidence supports at most a modest adjunctive role for HMB in exercise-induced metabolic adaptation rather than a strong independent anti-adiposity effect (Stout et al., 2015).

### 6.2 Preclinical evidence

A preclinical study evaluated the effects of combined HMB and *β*-alanine (β-Ala) supplementation on muscle function in aged Sprague–Dawley rats undergoing voluntary exercise. Twenty-month-old rats were provided with running wheels and assigned to either a control diet or a diet supplemented with HMB and β-Ala for 4 weeks. Supplementation did not significantly improve muscle force or fatigue resistance, although a modest increase in muscle cross-sectional area was observed. Consequently, MQ (force relative to size) declined in the supplemented group. Molecular analyses demonstrated reductions in markers of mitochondrial biogenesis (PGC-1α) and autophagy, together with alterations in myostatin signaling. Furthermore, voluntary physical activity increased only in control animals. These findings indicate that combined supplementation did not consistently enhance mitochondrial or functional adaptations and may even attenuate certain exercise-associated metabolic responses in aged muscle. Importantly, because HMB was co-administered with β-Ala, the independent contribution of HMB cannot be determined (Russ et al., 2017). Another study examined whether HMB supplementation could augment resistance training-induced hypertrophy and recovery in aged female rats. Nineteen-month-old rats were assigned to resistance training with HMB, resistance training alone, or control groups for 10 weeks. Resistance training significantly improved strength, grip performance, lean body mass, and reduced fat mass. Both resistance training groups also demonstrated increases in muscle fiber cross-sectional area, satellite cell number, and myonuclear content, particularly in the soleus muscle. Although HMB supplementation increased IGF-I mRNA expression, other myogenic markers, including mechano-growth factor and myogenin, increased similarly in both resistance training groups. These results suggest that resistance training was the principal driver of hypertrophic and regenerative adaptations, whereas HMB provided limited additional benefit beyond exercise-induced effects (Kim et al., 2012). Another investigation explored the combined effects of exercise, HMB, and black ginger in senescence-accelerated prone 8 mice, a model of age-related muscle decline. Aging was associated with reductions in muscle mass and strength, whereas exercise alone or combined with either HMB or black ginger produced limited improvements. In contrast, the triple intervention combining exercise, HMB, and black ginger significantly improved muscle mass and strength. Molecular analyses revealed upregulation of genes associated with mitochondrial biogenesis and energy metabolism, alongside modulation of autophagy-related proteins including Atg3, Atg7, Atg16L1, and Beclin1. However, because the beneficial effects were observed only with the multi-component intervention, attribution of these molecular and functional adaptations specifically to HMB remains difficult. The findings instead support the possibility that HMB may contribute adjunctively within broader multimodal anti-aging interventions rather than exerting robust independent effects (Aoki et al., 2022).

### 6.3 Critical synthesis of current evidence

The cumulative body of evidence pertaining to HMB-mediated neuroimmune, inflammatory, autophagic, and apoptotic regulation exhibits considerable heterogeneity and remains mechanistically incomplete. Although numerous investigations indicate enhancements in muscle function, inflammatory biomarkers, or molecular signaling pathways subsequent to HMB supplementation, these effects are often modest, contextually dependent, and inconsistently correlated with quantifiable alterations in muscle mass or body composition. Significantly, resistance training and multimodal exercise interventions consistently emerge as the predominant catalysts for functional and metabolic improvements, whereas the autonomous contribution of HMB frequently proves challenging to delineate, particularly in studies incorporating co-supplementation strategies or multicomponent interventions. Moreover, various documented molecular alterations including modifications in inflammatory gene expression, autophagy-related proteins, or mitochondrial biomarkers, exhibit ambiguous translational significance, as direct correlations between these molecular endpoints and clinically relevant functional outcomes remain inadequately substantiated. The existing corpus of evidence is further constrained by insufficient sample sizes, brief durations of intervention, diverse exercise protocols, varying participant characteristics, and significant methodological variability across both clinical and preclinical investigations. A considerable proportion of mechanistic insights is also derived from rodent models or analyses of peripheral skeletal muscle, which consequently limits the direct extrapolation to the biology of human aging or processes related to the CNS. In summary, the prevailing findings suggest a potential modulatory function for HMB in the regulation of peripheral inflammation, immune signaling associated with muscle, and pathways related to cellular stress responses during the aging process. Nonetheless, the current evidence does not yet confirm robust or consistent effects on neuroimmune regulation or specific inflammatory mechanisms within the CNS. Therefore, translational interpretations should be approached with caution until further investigations that are larger in scale, methodologically rigorous, and focused on the brain are conducted.

### 6.4 Molecular pathways of autophagy, apoptosis, and inflammatory modulation

Collectively, current evidence suggests that HMB may influence several molecular pathways involved in muscle maintenance, metabolic regulation, and exercise adaptation during aging; however, these effects appear to be modest, heterogeneous, and highly dependent on co-interventions such as resistance training, multimodal exercise, or additional nutritional compounds. HMB supplementation has been associated with alterations in markers related to mitochondrial function, autophagy, and apoptosis, including DRP1, MFN2, Beclin1, and members of the Atg signaling family. Some studies also reported reductions in apoptosis-related proteins such as Bax and cleaved caspases, suggesting potential anti-catabolic effects during periods of disuse or metabolic stress. Nevertheless, many of these molecular adaptations are also well-established consequences of exercise itself, making it difficult to determine the independent mechanistic contribution of HMB. Several investigations further demonstrated modulation of immune-related pathways, particularly involving inflammatory gene expression in T cells and cytokine-associated signaling molecules such as *IL-7R*, *LEF1*, and *RASGRP1*. These molecular changes correlated with improvements in muscle strength and functional performance. However, because these studies frequently combined HMB with exercise or multi-nutrient interventions, causal attribution to HMB alone remains limited. Some evidence additionally suggests that HMB may contribute to improvements in body composition and adiposity when combined with exercise interventions, although these effects are inconsistent and generally modest. Overall, the available literature primarily supports a peripheral role for HMB in modulating immunometabolic, anti-catabolic, and exercise-associated pathways related to skeletal muscle adaptation. Current evidence does not establish direct CNS-specific actions of HMB on neuronal signaling, synaptic plasticity, microglial regulation, or neurodegenerative pathology. Therefore, proposed links between HMB and brain–muscle–immune communication should presently be regarded as conceptual and indirect rather than mechanistically validated neurobiological pathways.

## 7 The brain–muscle–immune axis: a novel integrative framework

### 7.1 Proposed mechanistic framework of the brain–muscle–immune axis

The notion of a “brain–muscle–immune axis” has arisen as a comprehensive paradigm proposing a bidirectional communicative relationship among skeletal muscle, systemic immune modulation, metabolic processes, and the functionality of the CNS as it pertains to the aging process (Pedersen, 2019; Mattson, 2012; Delezie and Handschin, 2018; Gomes da Silva and Arida, 2015). Consequently, it is essential to experimentally differentiate the established peripheral effects of HMB from speculative neurobiological inferences. The prevailing body of evidence most robustly endorses the function of HMB in the peripheral regulation of musculoskeletal and metabolic systems. Experimental and clinical research illustrates that HMB may augment muscle protein synthesis (MPS), mitigate proteolysis, modulate mitochondrial-associated proteins, diminish apoptosis-related signaling, and facilitate functional adaptations in the context of resistance exercise and the aging process. Furthermore, several studies suggest that HMB may affect inflammatory gene expression, oxidative stress pathways, and cellular metabolic equilibrium, especially when synergistically combined with physical exercise interventions. Importantly, many of these physiological adaptations are also strongly mediated by exercise itself, thereby complicating attribution of independent HMB-specific effects. These empirical observations bolster the established function of HMB in the realms of peripheral immunometabolism and adaptations associated with physical exertion. A secondary mechanistic dimension encompasses systemic signaling cascades that likely interlink skeletal muscle activity with immune and neuroregulatory mechanisms. Skeletal muscle is increasingly acknowledged as an endocrine organ, proficient in the secretion of myokines, cytokines, metabolites, and extracellular signaling entities that may exert influence over systemic inflammatory equilibrium and remote organs, including the CNS (Severinsen and Pedersen, 2020; Eckel, 2019). Adaptations in muscle elicited by exercise can modulate the levels of circulating inflammatory mediators, enhance mitochondrial functionality, augment vascular performance, and regulate peripheral kynurenine metabolism via PGC-1α–dependent pathways (Handschin and Spiegelman, 2008). Notably, the metabolism of skeletal muscle associated with exercise may promote the peripheral conversion of kynurenine to kynurenic acid, thereby potentially diminishing the accumulation of neurotoxic kynurenine metabolites within the CNS (Agudelo et al., 2014). Although HMB has been associated with modulation of several exercise-related metabolic and inflammatory pathways, direct evidence demonstrating that HMB independently regulates kynurenine metabolism, neuroimmune signaling, or CNS-specific communication pathways remains unavailable.

Nevertheless, the shift from peripheral immunometabolic regulation to direct CNS-specific effects remains predominantly speculative. Although systemic reductions in inflammation, oxidative stress, metabolic dysfunction, and vascular impairment may theoretically promote healthy brain aging, no extant empirical evidence definitively establishes that HMB directly influences neuronal signaling, synaptic plasticity, neurotransmitter systems, microglial activation, or disease-specific neuropathology. Similarly, it remains ambiguous whether HMB traverses the BBB in biologically significant concentrations or whether neuronal and glial uptake occurs. Consequently, numerous proposed interactions between HMB supplementation and brain aging presently depend on indirect conceptual correlations rather than experimentally corroborated neurobiological mechanisms. Importantly, aging itself is progressively characterized by chronic low-grade inflammation (“inflammaging”), mitochondrial dysfunction, immune dysregulation, vascular impairment, and diminished metabolic flexibility, all of which contribute to both sarcopenia and neurodegenerative susceptibility (Franceschi et al., 2018; López-Otín et al., 2013; Hou et al., 2019). Exercise interventions are widely recognized for their capacity to enhance various systemic processes, and they have been shown to yield advantageous effects on cognitive function, vascular health, and the regulation of neuroinflammation (Erickson et al., 2019; Lourenco et al., 2019). Given that HMB is often administered concurrently with exercise interventions, many of the observed enhancements in cognitive or functional outcomes may primarily reflect physiological adaptations driven by exercise, rather than the independent neuroactive effects of HMB. Consequently, the extant literature supports a model in which HMB predominantly operates through peripheral metabolic, mitochondrial, inflammatory, and musculoskeletal pathways, which may indirectly affect the aging of the brain via systemic immunometabolic regulation. Nevertheless, the direct actions of HMB within the CNS remain inadequately characterized. Thus, the proposed brain–muscle–immune axis should currently be viewed as an evolving translational framework that necessitates considerable mechanistic validation, rather than as an established causal neurobiological pathway. Future investigations ought to encompass longitudinal mechanistic studies that integrate neuroimaging techniques, cerebrospinal fluid biomarker assessment, neuroimmune phenotyping, mitochondrial investigations, evaluations of the BBB, and multi-omics approaches. Furthermore, disease-specific experimental models targeting AD, PD, depression, and cognitive aging will be essential to ascertain whether the peripheral immunometabolic adaptations associated with HMB supplementation yield biologically significant downstream effects on the structure and functioning of the CNS.

### 7.2 Clinical evidence

A randomized, double-blind, placebo-controlled crossover investigation assessed the ramifications of a six-week regimen involving combined supplementation of creatine monohydrate (CRE) and HMB in conjunction with multicomponent exercise among physically active older adults (≥60 years). A total of thirty participants engaged in two distinct 6-week intervention phases, which were separated by a 3-week washout period. The combined administration of CRE and HMB markedly enhanced functional performance metrics, including gait velocity, sit-to-stand transitions, Timed Up and Go assessments, and 400-meter walking trials, whilst concurrently augmenting basal metabolic rate and overall metabolic efficiency. However, because the protocol simultaneously incorporated creatine supplementation and structured exercise training, the independent contribution of HMB cannot be clearly distinguished. Both exercise and creatine are well-established modulators of neuromuscular function, mitochondrial adaptation, and metabolic performance. No significant carryover effects were identified. Overall, the intervention appeared safe and was associated with improved mobility and metabolic function in older adults (Ramos-Hernández et al., 2026a) (Table 2). Another randomized, double-blind, placebo-controlled crossover study investigated the impacts of a six-week regimen of CRE in conjunction with HMB supplementation, integrated with a comprehensive physical conditioning (IPC) program among physically active older adults aged 60 years and above. A total of thirty subjects completed two distinct 6-week intervention phases, interspersed with a 3-week washout period. The combination of CRE and HMB alongside IPC yielded significant enhancements in functional strength, encompassing improvements in leg and back strength, arm flexion, upper-body endurance, and core endurance. Trends in body composition indicated a reduction in fat mass alongside marginal increases in muscular parameters; however, the variations observed within groups did not achieve statistical significance. Regression analyses revealed that the observed functional enhancements were predominantly independent of alterations in muscle mass, implying the occurrence of neuromuscular adaptations. Nevertheless, interpretation of HMB-specific effects remains limited because the intervention included both creatine and structured exercise, which themselves strongly influence neuromuscular performance and metabolic adaptation (Ramos-Hernández et al., 2025).

**Table 2:** Preclinical and clinical evidence on HMB and exercise interventions: Muscle, metabolic, and functional outcomes.

Population/model	Intervention	HMB formulation	Duration	Key outcomes	Findings	Reference
Clinical studies						
Physically active older adults (≥60 yr)	Creatine + HMB + supervised multicomponent training	NA	6 weeks (crossover)	Functional performance (gait speed, TUG, sit-to-stand), metabolic rate, inflammation, cardiopulmonary markers	Significant improvements in mobility, metabolic efficiency, and physiological markers; sex-specific vascular and respiratory adaptations suggest systemic (muscle–immune–cardiometabolic) crosstalk.	Ramos-Hernández et al. (2026a)
Physically active older adults (≥60 yr)	Creatine + HMB + multicomponent exercise (strength, HIIT, endurance)	NA	6 weeks (crossover)	Functional strength, endurance, body composition	Improved functional strength and endurance independent of muscle mass; supports neuromuscular and possibly central (brain-mediated) adaptations rather than hypertrophy-driven effects.	Ramos-Hernández et al. (2025)
Older adults with sarcopenia after hospitalization	HMB (3 g/day) + resistance training	Ca-HMB	12 weeks (+1-year follow-up)	Handgrip strength, SPPB, gait speed	Significant improvements in strength and physical performance, especially in women; sustained effects suggest interaction between muscle recovery, systemic inflammation, and functional capacity.	Meza-Valderrama et al. (2024)
Healthy older men (≈68 yr)	HMB-FA (3 g/day) + unilateral progressive RET	HMB-FA	6 weeks	Muscle protein synthesis, thigh lean mass, VL thickness, strength (1-RM, MVC)	RET induced strength gains in both groups; HMB-FA increased thigh lean mass and early MPS, but did not further enhance strength or VL hypertrophy; may confer long-term benefit.	Din et al. (2019)
Physically active older adults (≈63 yr)	Creatine + HMB + supervised exercise	NA	6 weeks (crossover)	Oxidized glutathione, Glutathione Redox Index, functional tests	Supplementation nominally preserved glutathione redox balance; weak exploratory associations with functional measures; supports potential antioxidant–neuromuscular crosstalk.	Ramos-Hernández et al. (2026b)
Older adults (≥65 yr) undergoing cardiac surgery	HMB 1.2 g + L-glutamine 7 g + L-arginine 7 g/day	NA	≥2 weeks pre-surgery	6-min walk distance (6MWD), muscle strength, physical function, hospital stay	Significant improvements in 6MWD pre- and post-surgery; enhanced strength and physical performance; shorter hospital stay; no effect on muscle mass; safe and feasible preoperative strategy.	Ogawa et al. (2025)
Institutionalized adults (83 ± 10 yr.; 63% women)	Vivifrail multicomponent exercise ± HMB 3 g/day	NA	12 weeks	Cognitive function, SPPB, handgrip, sit-to-stand power	Exercise improved cognitive and physical function and muscle power; HMB did not add further benefits; emphasizes importance of tailored exercise in very old adults.	Gutiérrez-Reguero et al. (2024)
Women (53 ± 1 yr)	HMB 3 g + Vitamin D3 2000 IU/day ± sedentary or RET	NA	12 weeks	Muscle volume, IMAT, lean mass, function	HMB + D reduced intermuscular adipose tissue (IMAT) and preserved arm lean mass in sedentary women; minimal effects on muscle function; highlights potential synergy with Vitamin D for muscle quality.	Fairfield et al. (2022)
Sarcopenic adults undergoing major GI surgery	HP ONS with HMB + resistance exercise + multimodal prehabilitation	NA	2–4 weeks pre-op	IMAT index, gait speed, anthropometrics, functional measures	Feasible prehabilitation with HMB improved gait speed and functional measures; increased IMAT index likely due to altered muscle metabolism; supports integration of nutritional supplementation with exercise in sarcopenic surgical patients.	Koh et al. (2024)
Preclinical studies						
Aged rats (34 mo)	Ca-HMB (340 mg/kg) ± hindlimb suspension/reloading	NA	2–4 weeks	Muscle fiber CSA, myonuclear apoptosis, caspase activity, maximal force	HMB attenuated fiber atrophy and apoptosis after disuse; reduced Bax and cleaved caspase-3/9, protecting against muscle loss via inhibition of mitochondrial-mediated myonuclear apoptosis.	Hao et al. (2011)

HMB, β-Hydroxy-β-Methylbutyrate; HMB-FA, Free acid form of HMB; CRE, Creatine monohydrate; RET, Resistance exercise training; IPC, Integral physical conditioning; EX, Exercise; CT, Control; SED, Sedentary; DXA, Dual-energy X-ray absorptiometry; MVC, Maximal voluntary contraction; 1-RM, One-repetition maximum; TLM, Thigh lean mass; VL, Vastus lateralis; TUG, Timed Up and Go; AFM, Abdominal fat mass; EPCR, Endothelial protein C receptor; SAMP8, Senescence-accelerated mouse-prone 8; BG, Black ginger; BMD, Bone mineral density. NA, Not Available.

Another study employed a randomized, double-blind, placebo-controlled methodology to assess the impact of 3 g/day HMB supplementation in conjunction with a 12-week RT regimen among older adults experiencing sarcopenia subsequent to post-acute hospitalization. The cohort receiving the intervention exhibited statistically significant enhancements in physical performance metrics, encompassing the total score of the Short Physical Performance Battery (SPPB), as well as improvements in balance and chair stand capabilities, when juxtaposed with baseline measurements. Notably, handgrip strength exhibited a marked increase in female participants, which was sustained at the 1-year follow-up assessment. These results indicated that calcium-bound HMB supplementation, when integrated with resistance training, has the potential to augment muscle strength and functional performance in elderly individuals recuperating from hospitalization. However, because all participants underwent resistance training, which is a primary driver of neuromuscular adaptation and functional recovery, the additional contribution of HMB appears modest and context dependent rather than independently causal. Furthermore, the relatively small sample size and rehabilitation setting limit broader mechanistic interpretation (Meza-Valderrama et al., 2024). Another investigation assessed 6 weeks of HMB free acid (HMB-FA) supplementation during unilateral resistance exercise training in healthy older men. Both HMB-FA and placebo groups improved muscle strength, knee extensor 1-RM, and vastus lateralis thickness following resistance exercise training. Although thigh lean mass increased significantly only in the HMB-FA group and was associated with transient early elevations in MPS, no significant differences were observed between groups for sustained MPS, myogenic gene expression, or hypertrophy- and atrophy-related molecular markers. Functional strength gains were also comparable between groups. These findings indicate that resistance exercise training itself accounted for most functional and muscular adaptations, whereas HMB-FA may have provided only limited adjunctive effects on lean mass during the early stages of training (Din et al., 2019).

### 7.3 Preclinical evidence

Another investigation examined the impact of HMB on muscle recuperation subsequent to disuse in aged rats. Following a duration of 14 days characterized by hindlimb suspension and the ensuing reloading phase, HMB administration was found to sustain maximal isometric force and enhance the cross-sectional area of plantaris and soleus fibers in comparison to control subjects. HMB treatment was associated with a reduction in myonuclear apoptosis, as evidenced by a diminished number of TUNEL-positive nuclei, a decrease in Bax protein levels, as well as reduced concentrations of cleaved caspase-3 and caspase-9, with Bcl-2 levels remaining unchanged. Although HMB was not entirely effective in preventing unloading-induced muscle atrophy, it did mitigate fiber loss in both fast-twitch and slow-twitch muscles. These results indicate that HMB facilitates muscle recovery primarily through the modulation of mitochondrial-associated apoptotic signaling pathways, the reduction of cellular apoptosis, and the promotion of hypertrophic responses during the reloading phase following disuse in aged skeletal muscle (Hao et al., 2011). However, these findings derive exclusively from peripheral skeletal muscle analyses and should not be directly extrapolated to CNS-specific or neuropsychiatric mechanisms.

### 7.4 Critical synthesis of current evidence

The cumulative evidence presently available that supports the hypothesized brain–muscle–immune axis involving HMB is characterized as preliminary, indirect, and lacking in mechanistic completeness. The majority of human studies predominantly indicate enhancements in physical performance, mobility, strength, or metabolic parameters, rather than elucidating direct neurobiological or neuroimmune outcomes. Moreover, a substantial number of interventions integrate HMB with exercise, creatine, or other nutritional strategies, which complicates the ability to delineate the independent impact of HMB on the observed physiological adaptations. A significant and recurrent observation across numerous studies is that functional improvements often transpire independently of considerable increases in muscle mass, indicating that neuromuscular, metabolic, or anti-catabolic adaptations may play a more critical role than hypertrophy alone. Nevertheless, the molecular underpinnings of these adaptations remain inadequately characterized, and direct evidence establishing a connection between peripheral musculoskeletal modifications and CNS-specific outcomes is presently absent. The translational interpretation of preclinical findings necessitates a prudent approach. While studies conducted on rodent models indicate potential effects of HMB on apoptotic signaling pathways, mitochondrial regulation, and muscle recovery subsequent to disuse, these investigations are constrained by species-specific physiological differences, highly regulated experimental conditions, and dependence on peripheral skeletal muscle biomarkers. As a result, the extrapolation of these findings to human brain aging, neuroimmune regulation, or neuropsychiatric disorders remains a matter of conjecture. In summary, the current body of evidence supports the hypothesis that HMB may indirectly engage with systemic pathways pertinent to muscle–immune communication and exercise adaptation throughout the aging process. However, the proposed brain–muscle–immune framework should currently be viewed as a conceptual and exploratory model rather than an experimentally validated neurobiological mechanism. Comprehensive, mechanistically rigorous, and CNS-oriented studies will be essential to ascertain whether biologically significant interactions among HMB, systemic metabolism, immune regulation, and brain aging genuinely exist.

### 7.5 Molecular basis of brain–muscle–immune communication

Collectively, available evidence suggests that HMB may influence several molecular pathways involved in skeletal muscle maintenance, metabolic regulation, and exercise adaptation during aging. HMB has been associated with transient increases in markers of MPS, particularly during the early phases of resistance training, and may modestly support preservation of lean body mass under catabolic or disuse conditions. In addition, several studies reported alterations in mitochondrial-associated proteins and regulators of autophagy, suggesting possible effects on cellular energy metabolism and protein turnover. HMB has also been linked to reductions in apoptosis-related markers, including Bax, cleaved caspase-3/9, and TUNEL-positive nuclei, which may contribute to preservation of muscle fibers during recovery from unloading or metabolic stress. Furthermore, some investigations demonstrated modulation of inflammatory and immune-related signaling pathways, including alterations in T-cell-associated gene expression and cytokine-related pathways. However, these findings are frequently derived from studies combining HMB with RT, creatine, vitamin D, or multicomponent exercise programs, making attribution of the observed molecular adaptations specifically to HMB difficult. Importantly, many pathways discussed within the proposed brain–muscle–immune framework including mitochondrial remodeling, inflammatory regulation, oxidative stress adaptation, and neuromuscular signaling, are also strongly regulated by exercise itself. Therefore, current evidence supports HMB primarily as a potential adjunctive modulator of peripheral metabolic and immunological processes rather than as an established independent regulator of systemic neuroimmune communication. At present, the strongest evidence supports indirect peripheral effects of HMB on muscle metabolism, inflammatory balance, and exercise adaptation, which could theoretically influence brain aging through systemic immunometabolic interactions. Although emerging preclinical studies suggest possible interactions between HMB and neurobiological processes, including cognition, BBB transport, and neuroinflammatory signaling, direct mechanistic evidence demonstrating biologically meaningful CNS-specific actions remains limited. Specifically, evidence linking HMB to neuronal signaling, synaptic plasticity, microglial regulation, neurotransmitter systems, or disease-specific neuropathology is still preliminary and largely derived from experimental or exploratory models rather than validated clinical neuroscience studies.

Significantly, numerous nascent preclinical and *in vitro* studies furnish initial mechanistic corroboration for the hypothesized muscle–brain–immune paradigm. Experimental investigations conducted on rodent models have indicated that HMB supplementation may sustain working memory, enhance long-term potentiation within the hippocampus, mitigate age-associated deficits in spatial learning, and influence neuronal morphology in the medial prefrontal cortex. Further research has proposed potential implications for BBB-related transport mechanisms, neurite extension, macrophage functionality, peripheral immune signaling, and T-cell-mediated neuroinflammatory reactions in models of experimental autoimmune encephalomyelitis (EAE). Nonetheless, these observations predominantly remain at the preclinical stage and necessitate prudent interpretation, as their translational applicability to human brain aging and neuropsychiatric conditions has yet to be established.

## 8 Effects of HMB on cognitive function and brain health

### 8.1 Preclinical and clinical evidence of HMB on cognition and brain function

A randomized, double-blind, placebo-controlled crossover trial examined the impact of concurrent supplementation with creatine (3 g/day) and HMB (3 g/day) on oxidative stress and redox balance in a cohort of 30 physically active older adults over 6 weeks of supervised exercise. Supplementation appeared to mitigate elevations in oxidized glutathione and contributed to preservation of the Glutathione Redox Index; however, these effects did not remain statistically significant after false discovery rate correction. Collectively, the findings suggest that combined creatine and HMB supplementation during exercise may exert modest effects on redox homeostasis; however, the concomitant use of exercise and creatine prevents attribution of these effects specifically to HMB. Importantly, the study did not directly evaluate cognition or CNS-specific outcomes (Ramos-Hernández et al., 2026b) (Table 2). Another randomized controlled trial assessed the impact of preoperative HMB supplementation on postoperative outcomes in older adults undergoing cardiac surgery. Participants received HMB together with l-glutamine and l-arginine for at least 2 weeks before surgery. Compared with controls, the intervention group demonstrated improvements in 6-min walking distance, muscle strength, physical performance, and duration of hospitalization, despite no significant changes in muscle mass. These findings support a possible role for perioperative nutritional support in functional recovery; however, the independent contribution of HMB cannot be isolated because of the combined formulation. Furthermore, no neurocognitive or neuropsychiatric endpoints were assessed (Ogawa et al., 2025).

A cluster-randomized study evaluated the effects of HMB supplementation combined with multicomponent exercise on cognitive and physical outcomes in institutionalized older adults. Participants were allocated to exercise alone, HMB alone, combined exercise plus HMB, or control groups for 12 weeks. Both exercise-containing groups demonstrated significant improvements in cognitive performance, SPPB scores, and muscular power, whereas the HMB-only group showed no significant benefit. These findings indicate that exercise was likely the principal mediator of the observed cognitive and functional improvements, while evidence for independent neuroactive effects of HMB remained limited (Gutiérrez-Reguero et al., 2024). A study investigating combined HMB and vitamin D3 supplementation in middle-aged women with vitamin D insufficiency reported improvements in lean mass preservation and reductions in intermuscular adipose tissue, particularly among sedentary participants. However, muscle functionality did not significantly improve, and no additional benefits were observed during resistance training. Importantly, the co-administration of vitamin D3 limits interpretation of HMB-specific effects, and no CNS-related outcomes were evaluated (Fairfield et al., 2022). A pilot investigation assessed a multimodal prehabilitation strategy involving high-protein oral nutritional supplementation containing HMB combined with resistance training in sarcopenic surgical patients. Temporary improvements in gait velocity and muscle-related parameters were observed following intervention. However, these benefits diminished after surgery. Because the intervention incorporated resistance training, protein supplementation, and additional perioperative optimization measures, attribution of the observed effects specifically to HMB is not possible. Moreover, cognitive and mechanistic CNS-related outcomes were not investigated (Koh et al., 2024).

### 8.2 Critical synthesis of current evidence

The existing body of evidence regarding the potential impacts of HMB on cognitive function and cerebral well-being is, in aggregate, characterized by limitations, indirectness, and considerable heterogeneity. The majority of investigations predominantly focus on musculoskeletal, metabolic, oxidative stress, or functional outcomes, rather than on direct neurobiological measures. Furthermore, numerous interventions amalgamate HMB with exercise, creatine, vitamin D, amino acids, or multimodal rehabilitation protocols, thereby significantly constraining the capacity to discern the independent effect of HMB on cognitive or neurological results. A salient recurring observation across numerous studies is that enhancements in cognitive and functional abilities are primarily noted within intervention groups that incorporate exercise, whereas HMB supplementation in isolation typically yields modest or statistically insignificant effects. This trend implies that neurophysiological adaptations associated with exercise including enhanced vascular function, systemic metabolic homeostasis, and diminutions in inflammation or oxidative stress, are likely the principal mechanisms mediating the cognitive benefits observed. Consequently, HMB may serve predominantly as an adjunctive source of metabolic or nutritional support rather than acting as a direct neuroactive agent. The extant evidence corpus is further limited by insufficient sample sizes, abbreviated intervention durations, diverse participant demographics, and inadequate integration of standardized neurocognitive, neuroimaging, or mechanistic endpoints related to the CNS. In addition, numerous studies depend on surrogate biomarkers, including redox indices or inflammatory markers, the direct correlation of which with clinically significant brain outcomes is still ambiguous. In summary, the prevailing body of evidence fails to conclusively demonstrate direct effects of HMB on cognitive function, neuroprotection, or brain-specific molecular pathways. Rather, the existing literature more convincingly suggests that HMB might indirectly affect facets of healthy brain aging via peripheral metabolic, exercise-induced, and immunophysiological adaptations. Consequently, it is imperative that larger, methodologically robust, and CNS-centric studies be conducted prior to drawing definitive conclusions regarding the neurocognitive or neuropsychiatric implications of HMB.

### 8.3 Molecular mechanisms mediating HMB’S neuroprotective effects

Current evidence suggests that HMB primarily influences peripheral metabolic and musculoskeletal pathways rather than directly established CNS-specific mechanisms. HMB has been associated with increased markers of MPS and reduced proteolytic signaling, including downregulation of Bax and caspase-3/9 pathways and reductions in TUNEL-positive nuclei in skeletal muscle fibers. Some studies also report modulation of myogenic and mitochondrial regulatory pathways involved in muscle maintenance and recovery. Additional observations involving intramuscular adipose tissue, inflammatory mediators, and redox balance suggest that HMB may contribute to peripheral metabolic resilience during aging and sarcopenia. When administered alongside resistance training, creatine, or vitamin D3, HMB has been associated with improvements in muscle strength, physical performance, and functional outcomes, often without major changes in muscle mass. However, because many studies incorporated exercise or co-supplementation strategies, the independent contribution of HMB remains difficult to determine. Importantly, current evidence supporting direct neuroprotective actions of HMB remains limited. Most available studies evaluate peripheral outcomes rather than neuronal signaling, synaptic plasticity, neurotransmission, or disease-specific neuropathology. Therefore, existing data more strongly support indirect immunometabolic and neuromuscular mechanisms that could theoretically influence brain aging, rather than definitive CNS-specific effects.

### 8.4 Preclinical and mechanistic evidence for neuroimmune regulation and cognitive function

#### 8.4.1 Cognitive function, synaptic plasticity, and hippocampal signaling

While the predominant focus of research on HMB has been on its effects on skeletal muscle metabolism, a number of preclinical studies have investigated its potential effects on cognitive function, synaptic plasticity, and hippocampal physiology. In aged rodent models, prolonged HMB supplementation has been correlated with enhancements in spatial learning, working memory, and cognitive flexibility. Kougias et al. (2017) illustrated that HMB mitigated age-related deficits in performance within the Morris water maze, particularly among male rats, implying possible influences on hippocampal-dependent memory processes. Likewise, Hankosky et al. (2017) documented enhancements in working memory and cognitive flexibility in aged rats following HMB supplementation, suggesting potential modulation of executive and hippocampal functions. Munroe et al. (2020) additionally noted the preservation of cognitive performance in aged mice undergoing long-term HMB supplementation, reinforcing the hypothesis that HMB may mitigate certain facets of age-related cognitive decline. Mechanistic investigations have indicated that these phenomena may encompass the modulation of synaptic plasticity alongside neuronal signaling cascades. Barranco et al. (2022) provided empirical evidence that HMB supplementation significantly augmented hippocampal long-term potentiation (LTP) and enhanced cognitive performance in working memory tasks among rodent subjects, thereby implicating mechanisms related to synaptic plasticity in the observed cognitive enhancements. Complementary findings from Paidi et al. (2023) elucidated the role of peroxisome proliferator-activated receptor alpha as a prospective molecular target of HMB in murine models, wherein supplementation was found to enhance hippocampal functionality and memory-related outcomes via pathways linked to neuronal metabolic processes and synaptic regulation. Furthermore, *in vitro* investigations suggest that HMB may facilitate neurite outgrowth in Neuro2a neuronal cells, thereby indicating potential neurotrophic or neurodevelopmental effects under controlled experimental conditions (Salto et al., 2015). Supplementary neuroanatomical findings lend credence to the hypothesis regarding the potential structural influences of HMB on the aging brain. Kougias et al. (2016) illustrated that HMB mitigated age-associated dendritic deterioration in pyramidal neurons located in the medial prefrontal cortex of senescent rats, thereby suggesting a possible preservation of neuronal morphology throughout the aging process. In aggregate, these investigations imply that HMB may modulate cognitive and neuronal functions through mechanisms that encompass synaptic plasticity, neuronal energetics, and the maintenance of neuronal structure. However, the translational relevance of these observations remains ambiguous. The majority of the existing evidence is derived from limited preclinical studies employing aging rodent models under rigorously controlled experimental paradigms. Furthermore, the cognitive effects observed tend to be relatively modest and may be influenced by factors such as sex, duration of supplementation, or metabolic context. Notably, direct evidence establishing a connection between HMB and established neurodegenerative mechanisms including amyloid-β accumulation, tau pathology, synaptic degeneration, neuronal mitochondrial dysfunction, or glial senescence, remains exceedingly scarce. Consequently, current evidence supports only a preliminary and exploratory role for HMB in cognitive aging rather than a validated neurotherapeutic effect.

#### 8.4.2 Neuroimmune regulation and inflammatory signaling

Emerging empirical evidence further indicates that HMB may play a pivotal role in modulating systemic immune responses and inflammatory signaling pathways pertinent to neuroimmune communication. Numerous studies suggest that HMB significantly affects immune cell proliferation, cytokine synthesis, and the expression of inflammatory genes. Nunes et al. (2011) elucidated that HMB influenced the proliferation and cytokine secretion in human peripheral blood mononuclear cells, thereby implying direct immunomodulatory effects on circulating immune populations. In a similar vein, Peterson et al. (1999) documented enhanced growth and functional capacity of macrophages subsequent to HMB exposure *in vitro*, thereby indicating that HMB may exert an influence on innate immune activity. Recent empirical findings further substantiate the plausible interactions between HMB and the regulation of immune-mediated inflammatory responses. Coleman et al. (2021) discerned that the administration of HMB supplementation enhanced antitumor immune responses in an obesity-sensitive murine model of pancreatic ductal adenocarcinoma, in part through the modulation of immune functions associated with macrophages. In clinical environments, Gonzalez et al. (2014) documented that HMB-FA modified the expression of immune-related biomarkers, including complement receptor 3 and macrophage inflammatory protein-1β, subsequent to resistance exercise, indicating that HMB may play a role in the modulation of inflammatory signaling during physiological stress reactions. Notably, Sheinin et al. (2023) evidenced that HMB inhibited the progression of EAE in murine subjects, a widely employed model for multiple sclerosis, potentially via the regulation of T-cell-mediated neuroinflammatory processes. These results furnish initial evidence suggesting that HMB may exert an influence on immune pathways pertinent to neuroinflammation and CNS autoimmune pathology. Notwithstanding these findings, the mechanistic nexus between peripheral immune modulation and CNS-specific outcomes remains inadequately delineated. The majority of extant studies assess systemic inflammatory markers or peripheral immune cells, rather than direct neuroimmune endpoints within the CNS. Moreover, numerous investigations incorporate concurrent exercise interventions or amalgamated nutritional formulations, thereby complicating the task of isolating HMB-specific immunological effects from more generalized exercise-mediated adaptations. Consequently, although HMB seems to possess the capability to modulate peripheral inflammatory and immune-related pathways, direct evidence substantiating the regulation of microglial activation, astrocyte functionality, or disease-specific neuroinflammatory cascades in human subjects remains elusive.

#### 8.4.3 BBB transport and CNS accessibility

One significant and unresolved inquiry pertains to the capacity of HMB to penetrate the CNS in concentrations that are biologically significant. Higuchi et al. (2021) elucidated a proton-coupled transport mechanism for HMB within the human BBB endothelial cell line hCMEC/D3, indicating that the transport of HMB across endothelial cells associated with the BBB may indeed be mechanistically plausible. This discovery furnishes a critical theoretical foundation for the potential accessibility of HMB to the CNS. Furthermore, additional indirect corroboration for CNS-related activities is derived from experimental models that reveal effects on hippocampal signaling, neuronal morphology, and neuroimmune regulation. Nevertheless, direct *in vivo* evidence substantiating BBB penetration, regional cerebral accumulation, neuronal uptake, or pharmacologically pertinent CNS concentrations of HMB in human subjects remains conspicuously absent. Additionally, measurements of cerebrospinal fluid and neuroimaging assessments have yet to be systematically conducted. Consequently, despite the preliminary experimental results indicating that HMB may have the potential to engage with CNS-associated pathways, the existing evidence is inadequate to draw a definitive conclusion regarding the direct neuroactive effects of HMB in human subjects. Additional mechanistic research incorporating BBB transport assessments, neuroimaging techniques, cerebrospinal fluid evaluations, and neuronal metabolic profiling will be necessary to ascertain whether significant biological exposure within the CNS is achieved.

#### 8.4.4 Critical interpretation and translational limitations

Collectively, the existing preclinical and mechanistic findings indicate that HMB may exert an influence on various biological processes potentially pertinent to cognitive aging and neuroimmune regulation, encompassing synaptic plasticity, inflammatory signaling, neuronal morphology, and metabolic homeostasis. Nonetheless, significant limitations hinder the translational interpretation of these findings. Firstly, a majority of the mechanistic evidence is derived from rodent or *in vitro* models rather than from investigations rooted in human neuroscience. Secondly, several observed cognitive or neuroimmune effects manifest concurrently with exercise interventions, combined nutritional supplementation, or rigorously controlled experimental paradigms, thereby complicating the attribution of causality specifically to HMB. Thirdly, evidence pertaining to specific diseases remains limited, with few investigations directly addressing AD pathology, tau aggregation, Parkinsonian neurodegeneration, cerebrovascular dysfunction, glial senescence, or neuronal mitochondrial failure. Furthermore, standardized neurocognitive, neuroimaging, and neuroimmune biomarkers are largely absent from the prevailing clinical trials. Consequently, the extant body of literature substantiates a conceivable yet not fully corroborated function for HMB in the realms of systemic neuroimmune interaction and cognitive governance. At this juncture, HMB ought to be predominantly considered as a peripheral metabolic and immunomodulatory agent with prospective secondary consequences for cerebral aging, rather than as a definitively established neuroprotective or CNS-targeted therapeutic intervention.

## 9 HMB, exercise synergy, and endurance performance

### 9.1 Clinical evidence for HMB–exercise synergy in endurance

In a 12-week multicenter investigation, the integration of muscle-targeted oral nutritional supplementation (MT-ONS; protein, Ca-HMB, and vitamin D3) with structured exercise produced modest improvements in muscle health among older adults with sarcopenia. Significant improvements were observed in appendicular lean mass, skeletal muscle index, handgrip strength, walking speed, chair-stand performance, and SPPB scores. Quality of life and serum vitamin D levels also improved. However, because the intervention simultaneously included exercise, protein supplementation, and vitamin D3, the independent physiological contribution of HMB cannot be definitively determined. Therefore, these findings are more appropriately interpreted as evidence supporting a multimodal intervention strategy rather than isolated HMB-specific efficacy (Sun et al., 2025) (Table 3). In a multicenter intensive care unit investigation, resistance training, with or without HMB supplementation, improved six-minute walking distance, SPPB scores, Medical Research Council scores, and grip strength in critically ill individuals. Importantly, HMB supplementation alone did not significantly affect primary outcomes, suggesting that exercise and rehabilitation were the principal drivers of functional improvement. Additionally, muscle mass, quality of life, and mortality remained unchanged, indicating that short-term interventions primarily enhanced functional performance rather than inducing substantial hypertrophic adaptations. Collectively, these findings support a comparatively modest and adjunctive role for HMB relative to exercise-mediated neuromuscular adaptation (Wu et al., 2023).

**Table 3:** Clinical evidence for HMB and exercise in older adults: functional, muscular, and cognitive outcomes.

Population/model	Intervention	HMB formulation	Duration	Key outcomes	Findings	Reference
Older adults ≥65 y with sarcopenia (*n* = 110)	MT-ONS containing protein + 0.5 g Ca-HMB/sachet + 200 IU D3 + structured exercise (resistance, chair-based, home-based activity)	Ca-HMB	12 weeks	Muscle mass (BIA), SMI, handgrip strength, walking speed, chair-stand, SPPB, serum vitamin D, QoL	Combined MT-ONS + exercise modestly improved muscle mass, strength, function, and QoL; relative contributions of exercise vs. supplementation not determined	Sun et al. (2025)
Medical ICU patients (*n* = 112, adults with internal medical diagnoses)	Resistance training alone, HMB 3 g/day, combination, or standard care	NA	Entire hospitalization	6MWD, SPPB, muscle strength (MRC and grip), QoL, ICU-AW, mortality	Resistance training ± HMB improved physical function and muscle strength; HMB alone had no significant effect; no changes in muscle mass, QoL, or 60-day mortality	Wu et al. (2023)
Older women 65–79 y with low muscle mass (*n* = 156)	Exercise (resistance training) ± Ca-HMB 1.5 g/day or placebo	NA	12 weeks + 12-week observation	Muscle mass (SMI), strength (knee extensor, hip adductor), gait speed, TUG, sit-to-stand	Exercise improved functional performance and strength; HMB only slightly improved usual gait speed; no additive effects of HMB on other outcomes	Osuka et al. (2021)
Older adults ≥60 y with low 25OH-D (*n* = 117)	Ca-HMB + Vitamin D3 ± resistance exercise; control ± exercise	Ca-HMB	12 months	Lean body mass, knee extension peak torque, functional index (handgrip, Get Up, GUG)	HMB + D improved muscle strength and functional index in non-exercisers; no additive effect in exercisers; supports HMB + D benefits even without exercise	Rathmacher et al. (2020)
Adults ≥65 y (men and women)	Ca-HMB 3 g/day ± resistance exercise 3 d/wk.; placebo ± resistance exercise	Ca-HMB	24 weeks	Lean mass (DXA), leg extension/flexion (LE/LF), handgrip (HG), get-up-and-go (GUG), muscle quality (MQ)	Ca-HMB alone improved strength, lean mass, and muscle quality; resistance exercise improved body composition and functionality; combined effects not additive	Stout et al. (2013)
Patients with liver cirrhosis (*n* = 24)	HMB 3 g/day vs. placebo (sorbitol powder)	NA	12 weeks	Muscle function (chair stand, 6MWT), quadriceps thickness (US), Liver Frailty Index (LFI)	HMB significantly improved muscle function, quadriceps thickness, and reduced frailty; well tolerated with no adverse events	Lattanzi et al. (2021)

HMB, β-Hydroxy-β-Methylbutyrate; EX, Exercise; EX+HMB, Exercise plus HMB supplementation; CT, Control; MT-ONS, Muscle-targeted oral nutritional supplementation; BIA, Bioelectrical impedance analysis; SPPB, Short Physical Performance Battery; 6MWD, Six-Minute Walk Distance; RET, Resistance Exercise Training; SED, Sedentary; IMAT, Intermuscular Adipose Tissue; HP ONS, High-Protein Oral Nutritional Supplementation; AI, Artificial Intelligence; LM, Lean Mass; FM, Fat Mass; MQ, Muscle Quality; EQ-5D, EuroQol-5 Dimensions; LFI, Liver Frailty Index. NA, Not Available.

In another 12-week factorial investigation involving older women with low muscle mass, exercise significantly improved muscle strength, gait velocity, and functional performance, whereas HMB supplementation alone produced only minimal improvement in habitual gait velocity. No synergistic interactions between HMB and exercise were identified for muscle mass, strength, or physical performance outcomes. Most benefits regressed during follow-up, underscoring the importance of sustained exercise interventions. These findings further support exercise as the dominant physiological stimulus underlying functional adaptation, with limited evidence for substantial additive effects attributable to HMB (Osuka et al., 2021). In a 12-month study involving older adults with vitamin D deficiency, combined Ca-HMB and vitamin D₃ supplementation improved lean body mass, knee extension torque, and composite functional performance in sedentary participants. No additional benefit was observed among physically active individuals. However, because vitamin D₃ itself influences muscle physiology and functional performance, the specific contribution of HMB cannot be isolated. Accordingly, the observed benefits should be interpreted cautiously as combined nutritional effects rather than direct evidence of independent HMB efficacy (Rathmacher et al., 2020).

In a 24-week investigation, Ca-HMB supplementation was associated with improvements in muscle strength, lean body mass, and MQ among older adults, including sedentary participants. Resistance training, irrespective of Ca-HMB supplementation, produced substantially larger improvements in strength, lean mass, and functional performance, reinforcing exercise as the principal determinant of physiological adaptation. Although Ca-HMB alone demonstrated modest anabolic and anti-catabolic associations, its effects were substantially smaller than those induced by resistance training, indicating that HMB may serve primarily as a supportive adjunct rather than a substitute for exercise-mediated adaptation (Stout et al., 2013). In a 12-week pilot investigation, HMB supplementation was associated with improvements in muscle mass and functional performance among individuals with liver cirrhosis. Participants demonstrated increased quadriceps thickness, improved chair-stand and six-minute walk performance, and reduced frailty scores. However, interpretation remains limited by the pilot design, small sample size, and absence of mechanistic biomarkers capable of distinguishing direct HMB-specific effects from broader nutritional or clinical recovery processes. Therefore, although the findings support the feasibility and tolerability of HMB supplementation in sarcopenic cirrhosis, larger controlled studies remain necessary before definitive conclusions regarding efficacy can be established (Lattanzi et al., 2021).

### 9.2 Critical synthesis of current evidence

The current body of evidence assessing the synergistic effects of HMB and exercise on endurance and functional performance is characterized by heterogeneity and contextual dependence. Although numerous studies document enhancements in muscle strength, physical performance, mobility, or MQ as a result of HMB supplementation, these advantages are often modest and inconsistently correlated with substantial increases in muscle mass. Notably, exercise interventions consistently emerge as the primary catalyst for functional adaptation across the majority of studies. A prominent observation indicates that HMB supplementation tends to yield greater benefits in populations that are sedentary, frail, nutritionally compromised, or clinically vulnerable, whereas additive effects are frequently limited or non-existent in individuals who are physically active and already engaged in structured exercise training. This variability implies that baseline physiological status, nutritional state, and levels of exercise exposure have a significant impact on the responsiveness to HMB supplementation. The interpretation of the evidence is further complicated by significant methodological heterogeneity among studies, encompassing variations in HMB formulations (Ca-HMB versus HMB-FA), co-supplementation strategies (for instance, vitamin D, protein, creatine), exercise protocols, intervention durations, and participant demographics. Numerous studies also feature relatively limited sample sizes, pilot-study frameworks, or abbreviated intervention periods, which consequently restrict statistical power and the comparability of results across investigations. Moreover, many of the observed enhancements in functional performance manifest independently of substantial hypertrophic responses, indicating that neuromuscular, metabolic, or anti-catabolic mechanisms may play a more pivotal role than mere muscle mass increase. Nevertheless, the specific molecular pathways that underlie these adaptations remain inadequately elucidated. The extant evidence supports a potential adjunctive function for HMB in augmenting certain exercise-related and metabolic outcomes during the aging process, especially within populations predisposed to sarcopenia or functional deterioration. Nonetheless, the current findings do not uniformly exhibit pronounced synergistic effects beyond those elicited by exercise alone, and thus, translational interpretation should be approached with caution.

### 9.3 Molecular mechanisms of HMB–exercise interaction in muscle and energy metabolism

Across these investigations, HMB supplementation was primarily associated with modest improvements in muscle strength, physical performance, and functional outcomes, particularly when combined with exercise or multimodal nutritional interventions. From a mechanistic perspective, HMB has been proposed to support MPS through activation of the mTOR pathway while potentially attenuating proteolytic activity via modulation of ubiquitin–proteasome and caspase-mediated apoptotic signaling. Some studies also suggest possible effects on mitochondrial-related proteins and cellular energy regulation. Additional observations include reductions in intramuscular adipose tissue and modest modulation of redox-related biomarkers, although these findings remain inconsistent across studies. Although exercise remains the principal driver of functional and metabolic adaptation, HMB may provide supportive anti-catabolic and metabolic effects in conditions associated with sarcopenia, frailty, or reduced physical activity. Importantly, many reported benefits occurred in studies involving co-interventions such as resistance training, creatine, vitamin D, or combined nutritional support, limiting causal attribution specifically to HMB. Overall, current evidence more strongly supports peripheral immunometabolic and exercise-associated actions of HMB than direct CNS-specific or neuroprotective mechanisms. Although systemic improvements in inflammation, metabolism, and physical function could theoretically influence brain aging, direct evidence involving neuronal signaling, synaptic plasticity, or neurodegenerative pathology remains limited.

## 10 Potential neurobiological relevance of HMB to neurodegenerative and neuropsychiatric disorders

### 10.1 AD and cognitive aging

Although empirical evidence pertaining specifically to the CNS regarding HMB is currently sparse, numerous peripheral molecular pathways that are modulated by HMB and physical exercise intersect with mechanisms that are implicated in the processes of brain aging and neurodegenerative diseases. AD and cognitive aging are increasingly acknowledged as multifactorial phenomena that encompass mitochondrial dysfunction, oxidative stress, compromised proteostasis, chronic neuroinflammatory responses, synaptic degeneration, neurovascular dysfunction, glial senescence, and impairment of the BBB (Franceschi et al., 2018; Hou et al., 2019; Mattson and Arumugam, 2018; Wyss-Coray, 2016). The progressive aggregation of amyloid-*β* plaques along with hyperphosphorylated tau protein plays a significant role in neuronal dysfunction and synaptic decline, whereas the activation of microglia and astrocytes exacerbates inflammatory signaling and neuronal damage (Selkoe and Hardy, 2016; De Strooper and Karran, 2016; Wang et al., 2014; Sweeney et al., 2018; Heneka et al., 2014). Mitochondrial dysfunction is regarded as a pivotal characteristic of both normative brain aging and the pathology of AD, leading to compromised neuronal bioenergetics, the accumulation of reactive oxygen species, and increased susceptibility of synapses (Cenini and Voos, 2019). Several empirical investigations incorporated into the current review suggest that HMB may influence mitochondrial-associated proteins, apoptosis-related signaling pathways, oxidative stress mechanisms, and autophagy-related processes in peripheral tissues. Moreover, interventions related to exercise that involve HMB have been associated with enhancements in systemic metabolic efficiency, regulation of inflammation, and functionality of muscle. These systemic adaptations could theoretically impact processes pertinent to the maintenance of healthy brain aging via indirect immunometabolic and vascular mechanisms. However, there exist no existing clinical or mechanistic studies that directly substantiate the assertion that HMB influences amyloid-*β* accumulation, tau phosphorylation, synaptic plasticity, neuronal survival, or the integrity of the BBB in human subjects. Consequently, any suggested relevance of HMB to the neuropathology associated with AD currently remains speculative and should be interpreted with caution. The evidence presently accessible more robustly supports indirect systemic effects facilitated through peripheral metabolic and exercise-related adaptations as opposed to definitive neuroprotective actions specific to the CNS.

### 10.2 PD and mitochondrial neurodegeneration

PD is defined by the progressive deterioration of dopaminergic neurons situated in the substantia nigra and encompasses a multitude of interrelated pathological processes, such as mitochondrial respiratory dysfunction, aggregation of *α*-synuclein, oxidative stress, disrupted proteostasis, and chronic activation of neuroinflammation (Poewe et al., 2017; Bose and Beal, 2016; Hirsch and Hunot, 2009). Among the aforementioned mechanisms, mitochondrial dysfunction especially concerning the impairment of complex I, is regarded as a significant factor contributing to neuronal degeneration and motor dysfunction in PD (Schapira, 2011). Numerous peripheral studies examined in this review indicate that HMB may exert an influence on mitochondrial dynamics, apoptotic signaling pathways, regulation of oxidative stress, and maintenance of metabolic homeostasis in skeletal muscle tissue. Given that mitochondrial dysfunction and redox imbalance are also pivotal in the pathogenesis of PD, these findings provide a theoretical framework for exploring the potential of systemic metabolic modulation via HMB to indirectly affect neurodegenerative processes. Furthermore, exercise-related interventions yield well-documented advantages regarding mitochondrial efficiency, inflammatory signaling, and vascular health, which may additionally enhance systemic resilience throughout the aging process. Nevertheless, there exists a notable deficit in direct mechanistic evidence that explicitly correlates HMB supplementation with the preservation of dopaminergic neurons, the regulation of *α*-synuclein, the recuperation of neuronal mitochondria, or the modulation of neuroinflammation specific to disease states. To date, no investigations have systematically examined the effects of HMB within established models of PD or evaluated outcomes related to neuropathology within the CNS. As a result, the translational significance of HMB in relation to PD is presently regarded as preliminary and insufficiently elucidated.

### 10.3 Depression, kynurenine signaling, and neuroimmune regulation

Depression and age-related neuropsychiatric disorders are increasingly recognized to involve intricate interactions among systemic inflammation, mitochondrial dysfunction, immune activation, skeletal muscle metabolism, and altered neuroimmune communication (Miller and Raison, 2016; Dantzer et al., 2008). Notably, the dysregulation of the kynurenine pathway has emerged as a significant mechanistic connection between peripheral inflammation and the manifestation of depressive symptoms. Adaptations in skeletal muscle induced by exercise may enhance peripheral kynurenine metabolism, thereby mitigating the accumulation of neurotoxic kynurenine metabolites within the CNS and potentially contributing to resilience against stress (Pedersen, 2019; Agudelo et al., 2014). Beyond kynurenine-related mechanisms, depression has also been correlated with compromised neuroplasticity, altered signaling of brain-derived neurotrophic factor (BDNF), mitochondrial dysfunction, oxidative stress, and chronic low-grade inflammation (Schlaepfer and Bewernick, 2014; Castrén and Monteggia, 2021). A number of studies reviewed in the current discussion suggest that HMB supplementation may impact inflammatory signaling pathways, immune-related gene expression, redox homeostasis, and muscle metabolism, particularly when synergistically combined with physical exercise interventions. These systemic modifications may theoretically facilitate neuroimmune regulation and indirectly bolster cognitive and psychological resilience throughout the aging process. Nonetheless, extant evidence directly correlating HMB with depression-associated neurobiology remains scarce. No existing investigations definitively establish that HMB influences neurotransmitter systems, BDNF signaling pathways, synaptic plasticity, emotional regulation, or clinically significant neuropsychiatric outcomes independent of physical exercise. Significantly, numerous cognitive or functional enhancements reported in the current literature are likely primarily attributable to exercise-induced physiological adaptations rather than direct neuroactive impacts of HMB itself. Consequently, the suggested role of HMB in neuropsychiatric regulation should currently be considered as tentative and mechanistically unverified.

### 10.4 Brain–muscle–immune communication and translational limitations

An emerging conceptual framework within the field of aging neuroscience posits that the bidirectional communication among skeletal muscle, systemic immunity, and CNS plays a significant role in promoting healthy aging as well as in determining susceptibility to neurodegenerative diseases. Exercise-induced myokines, inflammatory mediators, metabolic substrates, and mitochondrial signaling molecules may collectively exert an influence on neurovascular integrity, neuroinflammation, neuronal metabolism, and cognitive functioning (Pedersen, 2019; López-Otín et al., 2013; Erickson et al., 2019; Verkhratsky et al., 2019; Iadecola, 2017). Given that HMB is frequently investigated in association with exercise interventions and appears to modulate peripheral metabolic and inflammatory pathways, it has been postulated that HMB may indirectly engage in muscle–brain–immune communication during the aging process. Nevertheless, considerable mechanistic uncertainties persist unresolved. Among the critical unanswered inquiries are whether HMB traverses the BBB in biologically significant concentrations, whether uptake occurs within neuronal or glial cells, and whether HMB exerts a direct effect on synaptic signaling, neurotransmitter metabolism, microglial activation states, or pathways specific to disease-related neuropathology. Furthermore, the majority of contemporary human studies predominantly assess musculoskeletal, metabolic, or functional outcomes rather than standardized neurocognitive, neuroimaging, cerebrospinal fluid, or neuropathological endpoints. Importantly, numerous mechanistic hypotheses articulated in the academic literature including potential interactions involving kynurenine signaling, neuroinflammation, mitochondrial regulation, and exercise-associated brain–muscle–immune communication, are currently substantiated primarily by indirect or preclinical evidence rather than unequivocal causal proofs. Consequently, the posited neurobiological significance of HMB should presently be construed within the parameters of an evolving and exploratory translational framework rather than as a well-established CNS-targeted therapeutic paradigm. Future research endeavors should therefore integrate disease-specific preclinical models of AD, PD, depression, and cognitive aging in conjunction with longitudinal translational clinical studies that encompass neuroimaging, neuroimmune phenotyping, mitochondrial evaluations, BBB biomarkers, cerebrospinal fluid examinations, and multi-omics methodologies. Such methodological approaches will be pivotal in ascertaining whether peripheral immunometabolic adaptations linked to HMB supplementation yield biologically significant downstream effects on cerebral aging and neuropsychiatric disorders.

## 11 Clinical implications in aging, sarcopenia, and neuropsychiatric disorders

### 11.1 Frailty and functional decline

The synergistic interactions between HMB supplementation and physical exercise have been the subject of increasing scholarly inquiry among older adults, aimed at mitigating age-related functional deterioration, sarcopenia, and associated health complications. Empirical research from clinical trials indicates that HMB, whether administered independently or in conjunction with RT or multicomponent exercise regimens, can lead to modest improvements in muscle mass, strength, and functional metrics, including gait velocity, chair-stand performance, and SPPB assessments. HMB supplementation appears to confer significant advantages particularly for sedentary or nutritionally compromised older individuals, while exercise continues to serve as the primary catalyst for improvement. Furthermore, existing evidence suggests that HMB may facilitate improvements in MQ and reductions in intermuscular adipose tissue, thereby potentially supporting musculoskeletal health (Peng et al., 2021). These intervention strategies may offer pragmatic approaches to mitigating sarcopenia-associated decline and could potentially contribute to reductions in falls, hospitalizations, and loss of autonomy. However, current evidence primarily supports peripheral musculoskeletal and metabolic benefits, whereas direct CNS-specific or neuropsychiatric effects of HMB remain insufficiently characterized in large-scale clinical populations.

### 11.2 Depression and cognitive impairment

Frailty and functional decline are increasingly recognized as conditions associated with both physical deterioration and cognitive vulnerability in aging populations (Trombetti et al., 2016). Empirical investigations involving geriatric populations indicate that supplementation with HMB, particularly when utilized in conjunction with resistance or multicomponent exercise regimens, can mitigate the deterioration of appendicular lean mass, skeletal muscle index, and handgrip strength. Enhancements in gait velocity, sit-to-stand functionality, and six-minute walk distance have also been reported, underscoring the potential utility of HMB in supporting functional autonomy. Although physical exercise remains the predominant factor driving functional improvements, HMB may provide supplementary benefits for individuals unable to fully engage in physical activity (Courel-Ibáñez et al., 2019). Multicenter trials involving both institutionalized and community-dwelling older adults consistently demonstrate that HMB is generally well tolerated and may contribute to favorable outcomes related to frailty, mobility, and quality of life, thereby supporting its potential role as an adjunctive intervention in sarcopenia management and healthy aging. Nevertheless, evidence directly linking HMB supplementation to improvements in depressive symptoms, cognitive impairment, or neuropsychiatric disorders remains limited and largely indirect. Most currently available studies suggest that any observed cognitive benefits are predominantly attributable to exercise interventions, with HMB potentially serving as an adjunctive metabolic support rather than a direct neuroactive compound.

### 11.3 Translational relevance

Emerging research indicates that HMB supplementation may exert indirect influences on neuropsychiatric and cognitive outcomes by facilitating improvements in physical functioning and systemic metabolic processes. In geriatric populations, structured exercise regimens, whether incorporating HMB or not, have been associated with improvements in cognitive performance and executive functioning (Courel-Ibáñez et al., 2019). For instance, multicomponent exercise interventions have been shown to improve scores on the SPPB and may also be associated with enhanced performance on cognitive assessments, whereas HMB supplementation alone appears to provide comparatively modest benefits. Improvements in muscle mass, strength, and mobility may indirectly support brain health through mechanisms such as increased neurotrophic signaling, improved cerebral perfusion, and attenuation of oxidative stress, thereby suggesting a possible relationship between musculoskeletal and cognitive function. Although several preclinical and exploratory clinical studies suggest potential interactions between HMB, neuroinflammation, oxidative stress regulation, and proposed brain–muscle–immune communication, direct mechanistic evidence involving BBB penetration, neuronal signaling, synaptic modulation, or disease-specific neuropathology remains limited. Accordingly, current evidence primarily supports a peripheral and systemic role for HMB in healthy aging, with possible downstream implications for brain health mediated through immunometabolic and exercise-associated pathways. Therefore, HMB-exercise interventions should presently be considered promising but still exploratory strategies within aging neuroscience and geriatric healthcare frameworks.

## 12 Limitations of current evidence and knowledge gaps

Despite the expanding corpus of scholarly literature pertaining to HMB, numerous significant constraints and knowledge deficiencies persist that inhibit the formulation of definitive conclusions regarding its mechanistic and translational significance. Inconsistencies among various studies pose a substantial challenge. Although a multitude of trials document enhancements in muscle strength, functional capacity, and physical performance, the impacts of HMB on muscle mass, metabolic indicators, and inflammatory profiles exhibit considerable variability. Certain investigations reveal noteworthy functional improvements irrespective of hypertrophy, while others indicate minimal or nonexistent additive advantages beyond exercise alone. Likewise, findings associated with metabolic efficiency, oxidative stress, and inflammatory signaling are not consistently reproduced, likely attributable to variations in population characteristics (e.g., healthy individuals versus sarcopenic versus clinical cohorts), duration of intervention, and co-supplementation methodologies (e.g., creatine, vitamin D, amino acids). A pivotal knowledge deficiency is the absence of robust CNS-focused mechanistic and clinical evidence. Although indirect and preliminary evidence suggests that HMB may exert indirect effects on cognitive function, neuroinflammation, systemic immune signaling, and brain health through peripheral metabolic and immunological pathways, the preponderance of research is confined to peripheral tissues, with a particular emphasis on skeletal muscle. Direct mechanistic evidence involving BBB permeability, neuronal uptake, neurotransmitter signaling, synaptic modulation, microglial regulation, and disease-specific neuropathology remains extremely limited. Furthermore, many proposed neurobiological mechanisms, including kynurenine pathway modulation and exercise-associated brain–muscle–immune communication, are currently supported primarily by indirect or preclinical observations rather than definitive causal evidence. As a result, the hypothesized function of HMB within the brain–muscle–immune axis is presently exploratory, incompletely characterized, and necessitates direct empirical substantiation. Furthermore, methodological constraints further obfuscate interpretation. Numerous studies are constrained by limited sample sizes, brief intervention durations, and a lack of uniformity in study design, which includes discrepancies in dosing regimens, exercise protocols, and outcome metrics. The absence of standardized biomarkers and inadequate integration of molecular endpoints such as gene expression, proteomics, and mitochondrial functionality diminishes comparability between studies. Additionally, there is a paucity of longitudinal, multi-omics, and brain-oriented investigations integrating neuroimaging, neurocognitive assessments, immune phenotyping, and molecular profiling to clarify intricate systemic interactions. Importantly, most currently available human studies primarily evaluate musculoskeletal or functional outcomes rather than direct neurological or neuropsychiatric endpoints, thereby limiting translational interpretation within aging neuroscience. Addressing these limitations will be essential to advance HMB research from predominantly peripheral and descriptive observations toward a more mechanistically rigorous and CNS-relevant framework applicable to aging neuroscience and age-related disorders. Many included studies employed multicomponent interventions involving resistance training, creatine, vitamin D, protein supplementation, or structured rehabilitation programs, thereby limiting the ability to isolate HMB-specific effects.

## 13 Future directions: toward translational molecular neurobiology

Advancing the domain of HMB research necessitates a paradigmatic transition from predominantly peripheral, exercise-oriented, and descriptive investigations toward integrative, mechanism-oriented explorations within a translational molecular neurobiology context. A principal objective is the adoption of multi-omics methodologies to more comprehensively delineate the systemic ramifications of HMB. The integration of transcriptomics, proteomics, metabolomics, and epigenomics may facilitate the identification of molecular signatures associated with HMB-related adaptations. For instance, existing evidence indicating modifications in inflammatory gene expression, mitochondrial protein profiles, and autophagy-related pathways underscores the need for systems-level investigations to clarify interconnected regulatory networks. Multi-omics approaches may further improve understanding of how HMB influences redox homeostasis, immune signaling, and metabolic flux across diverse tissues, thereby potentially revealing novel molecular targets. Equally significant is the advancement of rigorous CNS-oriented investigations designed to determine whether HMB exerts biologically meaningful direct neurobiological effects or instead influences brain health predominantly through indirect systemic and muscle-derived pathways. The existing body of knowledge is primarily derived from peripheral observations, with limited direct evidence regarding brain-specific mechanisms. Future investigations should therefore examine HMB’s potential ability to traverse the BBB, its possible interactions with neurotransmitter systems (including serotonin and kynurenine metabolism), and its putative roles in neuroinflammation, synaptic plasticity, neuronal viability, microglial activation, and BBB integrity. Both preclinical models and human neuroimaging or cerebrospinal fluid assessments will be important for determining whether CNS-specific effects of HMB can be experimentally validated in aging and neuropsychiatric contexts. Future experimental studies should also incorporate disease-relevant models of neurodegeneration, cognitive impairment, and neuroinflammation to better align mechanistic observations with translational neuroscience applications. Ultimately, the incorporation of personalized and precision medicine perspectives may improve the clinical relevance of HMB interventions. The variability in responses documented across studies suggests that factors such as age, sex, baseline nutritional status, physical activity level, and comorbidities substantially influence outcomes. Future trials may benefit from implementing stratified designs and biomarker-guided methodologies to identify responder profiles and optimize dosing regimens. The integration of genetic and metabolic profiling may further facilitate personalized interventions targeting specific neuro-metabolic phenotypes. Importantly, future randomized clinical trials should incorporate standardized neurocognitive, neuropsychiatric, inflammatory, and neuroimaging endpoints rather than relying predominantly on musculoskeletal or functional outcomes alone. Collectively, these future directions may help advance HMB research from heterogeneous and largely peripheral observations toward a more mechanistically rigorous and translationally relevant framework for understanding the potential interactions among HMB, systemic metabolism, exercise adaptation, and brain aging.

## 14 Conclusion

Current evidence supports the role of HMB primarily as a peripheral metabolic and immunometabolic modulator associated with improvements in muscle strength, physical performance, and selected functional outcomes, particularly among aging individuals and clinical populations. Nevertheless, findings regarding muscle mass, mitochondrial adaptations, inflammatory signaling, and long-term functional benefits remain heterogeneous and frequently inconsistent across studies. Importantly, many reported benefits occur in the context of resistance training, multicomponent exercise programs, creatine supplementation, vitamin D administration, or combined nutritional interventions, making it difficult to isolate the independent contribution of HMB from exercise-mediated or co-supplementation-mediated effects. The available literature suggests that HMB may influence peripheral molecular pathways involved in protein turnover, mitochondrial regulation, autophagy-related signaling, apoptosis-associated pathways, and inflammatory gene expression. However, the magnitude and reproducibility of these molecular effects vary substantially between experimental settings, populations, and intervention protocols. In many studies, exercise itself appears to be the dominant driver of neuromuscular, metabolic, and functional adaptation, whereas HMB demonstrates comparatively modest, context-dependent, or adjunctive effects. A growing area of interest concerns the potential relationship between peripheral metabolic adaptations and brain aging through proposed muscle–brain–immune interactions. Although certain studies report associations involving inflammatory signaling, oxidative stress modulation, immune-related gene expression, and functional or cognitive outcomes, direct mechanistic evidence linking HMB to CNS regulation remains limited. Proposed mechanisms involving kynurenine metabolism, neuroinflammation, synaptic plasticity, microglial signaling, BBB regulation, and neurotransmitter systems remain largely hypothetical and insufficiently validated experimentally. Importantly, the current evidence base is constrained by small sample sizes, heterogeneous study designs, short intervention durations, inconsistent outcome measures, and limited integration of mechanistic neuroscience endpoints. Most available human studies focus predominantly on musculoskeletal or exercise-related outcomes rather than direct neurobiological, neuroimaging, or neuropsychiatric assessments. Accordingly, HMB should presently be regarded primarily as a nutritional compound with potential supportive roles in peripheral metabolic regulation, exercise adaptation, and muscle preservation during aging, rather than as an established neuroprotective or CNS-active intervention. Future investigations should prioritize mechanistically rigorous, CNS-oriented, and longitudinal translational studies integrating neuroimaging, cerebrospinal fluid biomarkers, multi-omics profiling, immune phenotyping, and disease-specific models of neurodegeneration and cognitive aging to determine whether biologically meaningful links between HMB, systemic metabolism, and brain health truly exist. Recent preclinical investigations involving rodent, immune-cell, and BBB models provide emerging evidence that HMB may influence hippocampal plasticity, neuroimmune signaling, neuronal morphology, and peripheral immune regulation; however, the clinical significance and translational applicability of these observations remain uncertain.
